# The reduced activity of PP-1α under redox stress condition is a consequence of GSH-mediated transient disulfide formation

**DOI:** 10.1038/s41598-018-36267-6

**Published:** 2018-12-07

**Authors:** Simranjit Singh, Simon Lämmle, Heiko Giese, Susanne Kämmerer, Stefanie Meyer-Roxlau, Ezzaldin Ahmed Alfar, Hassan Dihazi, Kaomei Guan, Ali El-Armouche, Florian Richter

**Affiliations:** 1 Institute of Pharmacology and Toxicology, Technische Universität Dresden, Dresden, Germany; 2 Institute of Pharmacology and Toxicology, Universitätsmedizin Göttingen (UMG), Göttingen, Germany; 3 Molecular Bioinformatics, Goethe-Universität Frankfurt am Main, Frankfurt, Germany; 4 Clinic for Nephrology and Rheumatology, UMG, Göttingen, Germany; 5 Functional Proteomics at the Faculty of Medicine, Goethe-Universität Frankfurt am Main, Frankfurt, Germany

## Abstract

Heart failure is the most common cause of morbidity and hospitalization in the western civilization. Protein phosphatases play a key role in the basal cardiac contractility and in the responses to β-adrenergic stimulation with type-1 phosphatase (PP-1) being major contributor. We propose here that formation of transient disulfide bridges in PP-1α might play a leading role in oxidative stress response. First, we established an optimized workflow, the so-called “cross-over-read” search method, for the identification of disulfide-linked species using permutated databases. By applying this method, we demonstrate the formation of unexpected transient disulfides in PP-1α to shelter against over-oxidation. This protection mechanism strongly depends on the fast response in the presence of reduced glutathione. Our work points out that the dimerization of PP-1α involving Cys^39^ and Cys^127^ is presumably important for the protection of PP-1α active surface in the absence of a substrate. We finally give insight into the electron transport from the PP-1α catalytic core to the surface. Our data suggest that the formation of transient disulfides might be a general mechanism of proteins to escape from irreversible cysteine oxidation and to prevent their complete inactivation.

## Introduction

Post-translational modifications (PTMs) including phosphorylation, glycosylation, acetylation, hydroxylation, proteolytic cleavage as well as oxidative modifications are involved in the maintenance of the body homeostasis. In failing hearts, both abnormal phosphorylation states of essential cardiac proteins and elevated production of reactive oxygen species (ROS) contribute to contractile dysfunction of the heart^1–3^. During normal cellular aerobic function, hydrogen peroxide (H_2_O_2_), hydroxyl radical and superoxide, known as ROS, are produced in the cells^4^.

ROS serve as secondary messengers that control signal transduction by oxidizing the most sensitive cysteine (Cys) of various kinases and phosphatases^5–7^. In the heart, protein phosphatase type-1 (PP-1) and type-2 (PP-2) are the major constituents of phosphatase activity (~90%). PP-1 is ubiquitously expressed in most cardiac cell types, including cardiomyocytes^8,9^. Three isoforms of PP-1, α, β and γ, have different subcellular localizations and substrate binding patterns^10^. PP-1 harbours various PTMs including redox-PTMs that could play a major role in the control of its activity. Structural elements of PP-1 contributing to its activity regulation involve the metal centre (two Mn^2+^ ions being chelated by four His-residues), the substrate binding site and the regulatory element binding sites^11–13^. PP-1 activity is inhibited by H_2_O_2_ treatment both *in vitro* and *in vivo*, and this inhibition could be reversed *in vitro* by thiol-antioxidant *N*-acetyl-cysteine (NAC) or reduced glutathione (GSH)^14^. Moreover, PP-1α was associated with oxidative brain diseases such as schizophrenia^15^, alcohol abuse induced ciliary dysfunction (AICD)^16^. Interestingly, PP-1 was found as a target of peroxide stress in the moderately oxidized brains to form transient disulfides, which may promote the slow accrual of neuronal damage that could detour healthy brain development and aging^17^. However, the underlying mechanism of how redox stress controls PP-1 activity has not been well understood.

Here, we tested the hypothesis that the transient and dynamic disulfide formation might prevent over-oxidation and irreversible inactivation of PP-1α. First, we used elaborated mass spectrometry (MS) techniques and present an optimized workflow, the so-called “cross-over-read” method, for the identification of disulfide-linked peptides from MS^2^ spectra that can be performed on every high-resolution detection system. With this method, we could identify both intra- and inter-molecular disulfide bridges in superoxide dismutase 1 (SOD1) and pyruvate kinase M2 (PKM2). Moreover, we performed a simple combination of experiments by using recombinant PP-1α (rPP-1α) with and without glutathione S-transferase (GST) activity and analysed the formation of transient disulfides in PP-1α upon oxidative stress. We show that the establishment of disulfides in rPP-1α strongly depends on the fast reaction when GSH is present. Our data suggest that the formation of transient disulfide bridges might be a general mechanism of proteins to escape from over-oxidation.

## Results

### Impact of oxidative stress on the PP-1 activity and its downstream targets

**Figure 1:**
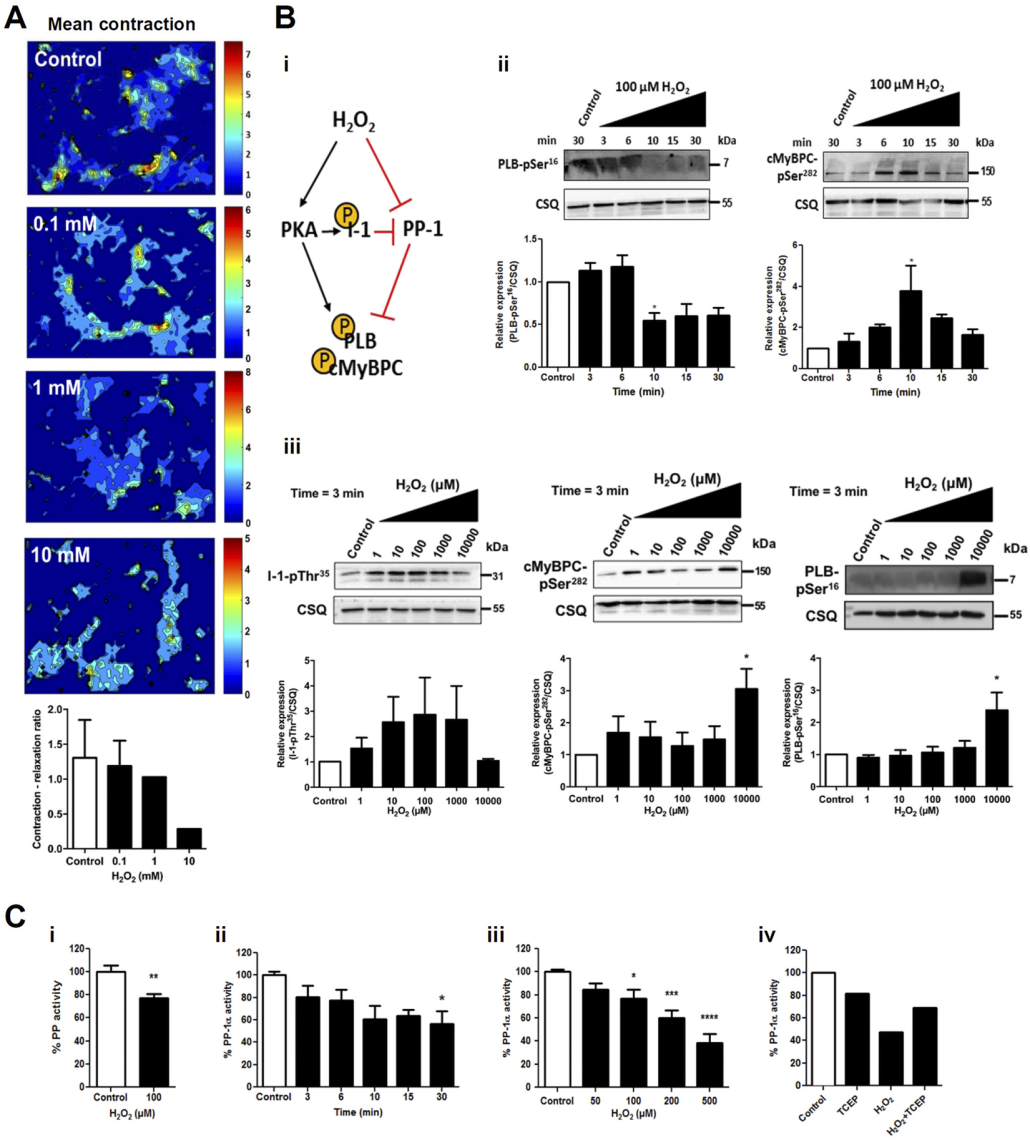
Effect of H_2_O_2_ treatment on cardiomyocyte survival, the PP-1α activity and phosphorylation of its downstream targets. (**A**) Pictures and quantification of NRCM mobility under oxidative stress from the time-lapse movie. (**B**) Quantitative analyses of immunoblots for downstream targets of PP-1α in NRCMs. (i) Mechanism of PKA activation and PP-1α inactivation upon H_2_O_2_ treatment. (ii) Time-dependent levels of PLB-pSer^16^ and cMyBPC-pSer^282^ after H_2_O_2_ treatment (n = 3). (iii) H_2_O_2_ concentration-dependent levels of I-1-pThr^35^, PLB-pSer^16^ and cMyBPC-pSer^282^ (n = 3). (**C**) Total phosphatase activity in NRCMs with and without H_2_O_2_ treatment (100 µM, 3 min, n = 5; i), time-dependent rPP-1α activity after H_2_O_2_ treatment (200 μM, n = 2-4; ii), H_2_O_2_ concentration-dependent rPP-1α activity (10 min, n = 4; iii) and recovery of rPP-1α activity (H_2_O_2_ 200 μM for 15 min; TCEP 100 mM for 5 min; iv). Data shown in panel Civ represent one experiment out of two independent experiments. Data are presented as mean ± SEM; *P < 0.05, **P < 0.01, ***P < 0.001 and ****P < 0.0001 using one-way ANOVA and Bonferroni’s correction.

First, we investigated the effect of increasing concentrations of H_2_O_2_ on contraction and survival of neonatal rat cardiomyocytes (NRCMs). Live imaging was performed and a matlab-based script, as described previously^18^, was used to generate a heat map of the mean contraction of the cells (Figs 1A, S1). The contraction-relaxation ratios as a measure of myocyte vitality were calculated (Fig. 1A). As expected, the contraction of NRCMs was normal at a concentration of 0.1 mM H_2_O_2_, similar to control NRCMs without H_2_O_2_ treatment. With concentrations higher than 1 mM, the contraction was reduced and at 10 mM cells were dying.

To understand the effect of oxidative stress on the PP-1α activity, we investigated the phosphorylation status of its downstream targets phospholamban (PLB) and cardiac myosin binding protein-C (cMyBPC) in NRCMs. We monitored phosphorylation of inhibitor-1 (I-1) that is responsible for the crosstalk between protein kinase A (PKA) and PP-1α signalling (Fig. 1Bi). We observed a slight increase in PLB phosphorylation at Ser^16^ (PLB-pSer^16^) in NRCMs treated with 100 µM H_2_O_2_ for 3–6 min and a remarkable decrease in PLB-pSer^16^ after 10 min. In contrast, changes in cMyBPC phosphorylation at Ser^282^ (cMyBPC-pSer^282^) showed a bell-shape, peaking after 10 min with 100 µM H_2_O_2_ (Fig. 1Bii). These data suggest different kinetics of PKA stimulation and PP-1 inhibition under H_2_O_2_ treatment, leading to the site-specific, temporal dynamics of phosphorylation of different substrates, as demonstrated previously^19^. By fixing the incubation time to 3 min and altering the concentrations of H_2_O_2_, we observed a bell-shaped response for I-1 phosphorylation at Thr^35^ (I-1-pThr^35^) with a peak at 100 μM, whereas PLB-pSer^16^ and cMyBPC-pSer^282^ were not changed much with 100 µM H_2_O_2_. This bell-shaped phosphorylation response of PKA substrates to the increased H_2_O_2_ concentrations was already described previously^5^. However, PLB-pSer^16^ and cMyBPC-pSer^282^ were increased at 10000 μM H_2_O_2_ (Fig. 1Biii), suggesting that PKA was still active while PP-1α was inhibited.

Next, we measured phosphatase activity in NRCMs and found the net decrease in phosphatase activity about 25% when we treated NRCMs with 100 µM H_2_O_2_ for 3 min (Fig. 1Ci). By using rPP-1α, we established a reproducible *in-vitro* phosphatase activity assay and observed a maximum effect of H_2_O_2_ treatment (200 µM) after 10 min (Fig. 1Cii). By applying increasing concentrations of H_2_O_2_, we showed a reduction of the rPP-1α activity by 50% at 500 µM H_2_O_2_ for 10 min (Fig. 1Ciii). Interestingly, the inhibition of rPP-1α activity by H_2_O_2_ (200 µM for 15 min) was partially rescued with the mild reducing agent Tris(2-carboxyethyl)phosphine (TCEP) at 100 mM for 5 min (Fig. 1Civ), suggesting that the inactivation of PP-1α by oxidation is reversible.

### In-gel dimerization detection of PP-1α

**Figure 2:**
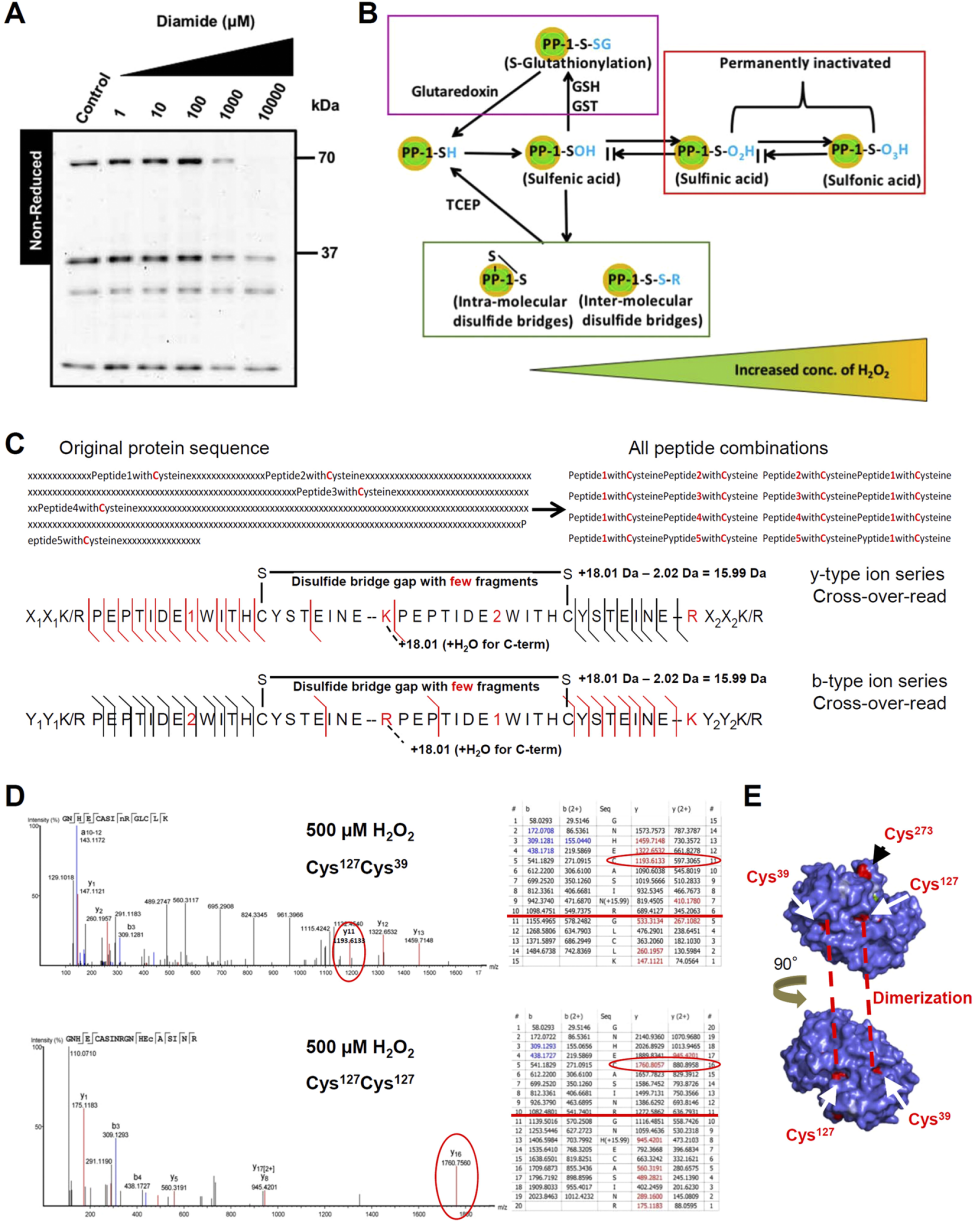
Dimerization of PP-1α involves disulfide formations. (**A**) Shown is the oxidative stress-independent dimerization of PP-1α in NRCMs in non-reducing PAGE. (**B**) General scheme of the sulfhydryl groups formed upon H_2_O_2_ treatment: reversible modifications including sulfenic acid, S-glutathionylation (violet box) and disulfides (green box) as well as irreversible modifications (sulfinic acid and sulfonic acid; red box). (**C**) A robust search strategy for the identification of disulfide bridges. When searching the spectra in a linear database, disulfide formation would not be detectable. Generation of permutated combination database and the “cross-over-read” mechanism with independent y- and b-type ion series identified from two database entries helps the identification of disulfide-linked MS/MS-spectra. Large molecular-weight fragments are indicated in red. (**D**) Spectra (left) and ion tables (right) for Cys^39^Cys^127^ and Cys^127^Cys^127^ disulfides identified at 500 µM H_2_O_2_ (y-type: red; b-type: blue). Red lines indicate the end of peptide1. (**E**) Cysteine interactions indicated with dashed lines between two 90°-turned monomers of PP-1α.

To decipher the mechanism responsible for the reversible inactivation of PP-1α, we investigated whether oxidative stress might induce PP-1α dimerization through surface cysteine modification in NRCMs. We first established non-reducing conditions suitable for the in-gel detection of protein dimerization by analysing PKA and PP-2Ac upon oxidative stress (Fig. S2A). We found an increase in dimerization of PKA at higher concentrations of diamide and H_2_O_2_ with consistently reduced monomer formation, but no dimerization of PP-2Ac. In contrast, we observed dimerization of PP-1α in NRCMs without oxidative stress and after treatment with diamide (Fig. 2A). Quantitative analysis showed that PP-1α monomer expression was already decreased at 1 µM diamide with further reduction up to 10 mM, whereas the dimer expression increased up to 100 µM diamide. At 1 mM diamide, both monomer and dimer expression of PP-1α were reduced (Fig. S2B). At the highest concentration of 10 mM diamide, we observed strong reduction of both PP-1α monomer and dimer due to severe cell damage. Therefore, the dimer/monomer ratio showed a bell-shaped curve peaking at 100 µM diamide (Fig. S2B). These data indicate that the dimerization of PP-1α might not be the primary cause for the reversible inactivation of PP-1α under oxidative stress.

### Establishment of new search strategy for detection of disulfide formation in PP-1α

Based on our in-gel data, we tested whether the reversible Cys-modifications in PP-1α occur under oxidative stress. As illustrated in Fig. 2B, the first step in cysteine oxidation is the formation of sulfenic acid and then different Cys-pools formed with increased concentration of H_2_O_2_: (i) reversible intra- and inter-molecular disulfides, (ii) reversible glutathionylation and (iii) irreversible sulfonic acid. PP-1α owns 13 Cys-residues and can be differently influenced by the solvent. By using the modified search strategy for chemically crosslinked peptides as previously published^20^, we could not get high-quality spectra.

For the identification of disulfide-linked peptides, we established a new database search strategy, which is based on sequence tag search in the PEAKS software. In brief, we applied the concept of searching against linearized databases containing all permutated combinations of Cys-peptides. The databases were either created manually or in an automated way for more complex datasets easily resulting in more than 100,000 combinations. More specificity was obtained by organizing every di-peptide as a separate protein entry and searching without enzyme specificity (Fig. 2C). We introduced a modification for the hidden C-terminus of peptide1 (+18 a.m.u.) to identify the spectra that matched the two database entries peptide1-peptide2 and peptide2-peptide1. The MS/MS ion series of one peptide stopped at the disulfide link and continued by the ion series of the other peptide in the higher molecular weight region of the spectrum. We hence termed the generation of composite spectrum “cross-over-read” (Fig. 2C).

By applying the new strategy, we searched for free Cys and disulfides in GST-tagged rPP-1α at increasing concentrations of H_2_O_2_ (0, 100, 500 µM) in the absence of Mn^2+^. We observed disulfides between Cys^39^ and Cys^127^ at all concentrations and one homo-di-peptide of Cys^127^ at 500 µM (Fig. 2D, Fig. S2C,D). Cys^39^ and Cys^127^ were not found as free cysteines at all concentrations (Fig. S2D). However, a direct link between Cys^39^ and Cys^127^ within one monomer of PP-1α is unlikely due to their molecular distance about 15.72 Å (Fig. S3). Because both of them are surface-oriented to the same side, the interaction of two monomers with each other being turned by 90° is possible (Fig. 2E). These data are consistent with the in-gel detection of PP-1α dimerization even without oxidative stress. It is known that the major substrate binding sites of PP-1α, for instance, for spinophilin^21^, include interactions of Cys^140^, Cys^127^, Cys^273^ and eventually Cys^202^ towards an acidic patch (Fig. S4). Our results suggest that the formation of inter-molecular disulfide bridges between Cys^39^ and Cys^127^ and between two Cys^127^ might protect the active surface of PP-1α when the substrate is absent.

### SOD1 as additional model for the detection of disulfide bridges

To validate if the “cross-over-read” method is suitable for detection of disulfides for other proteins, we analysed bovine SOD1 with only 3 Cys-residues Cys^7^, Cys^56^ and Cys^145^ (Fig. S5). As expected, we detected the intra-molecular disulfide bridge between Cys^56^ and Cys^145^ (Fig. S5A,C), which is essentially required for correct folding and metal ion binding^22^. Importantly, we observed disulfide formation between two copies of N-terminally acetylated Cys^7^ peptides and between two copies of Cys^145^ (Fig. S5A,C). The homo-dimer spectra could be unequivocally identified by high molecular weight fragments (Fig. S5C). We furthermore identified one mixed disulfide with a lower spectrum quality between Cys^7^ and Cys^56^ (Fig. S5A,C). In contrast, we could not identify any homo-di-peptides of Cys^56^ (Fig. S5B), which would have a similar probability due to the molecular distance (15.90 Å). These data indicate that the “cross-over-read” search strategy is suitable to detect both intra- and inter-molecular disulfide bridges.

### Transient disulfide formation in PP-1α is enhanced by S-glutathionylation

**Figure 3:**
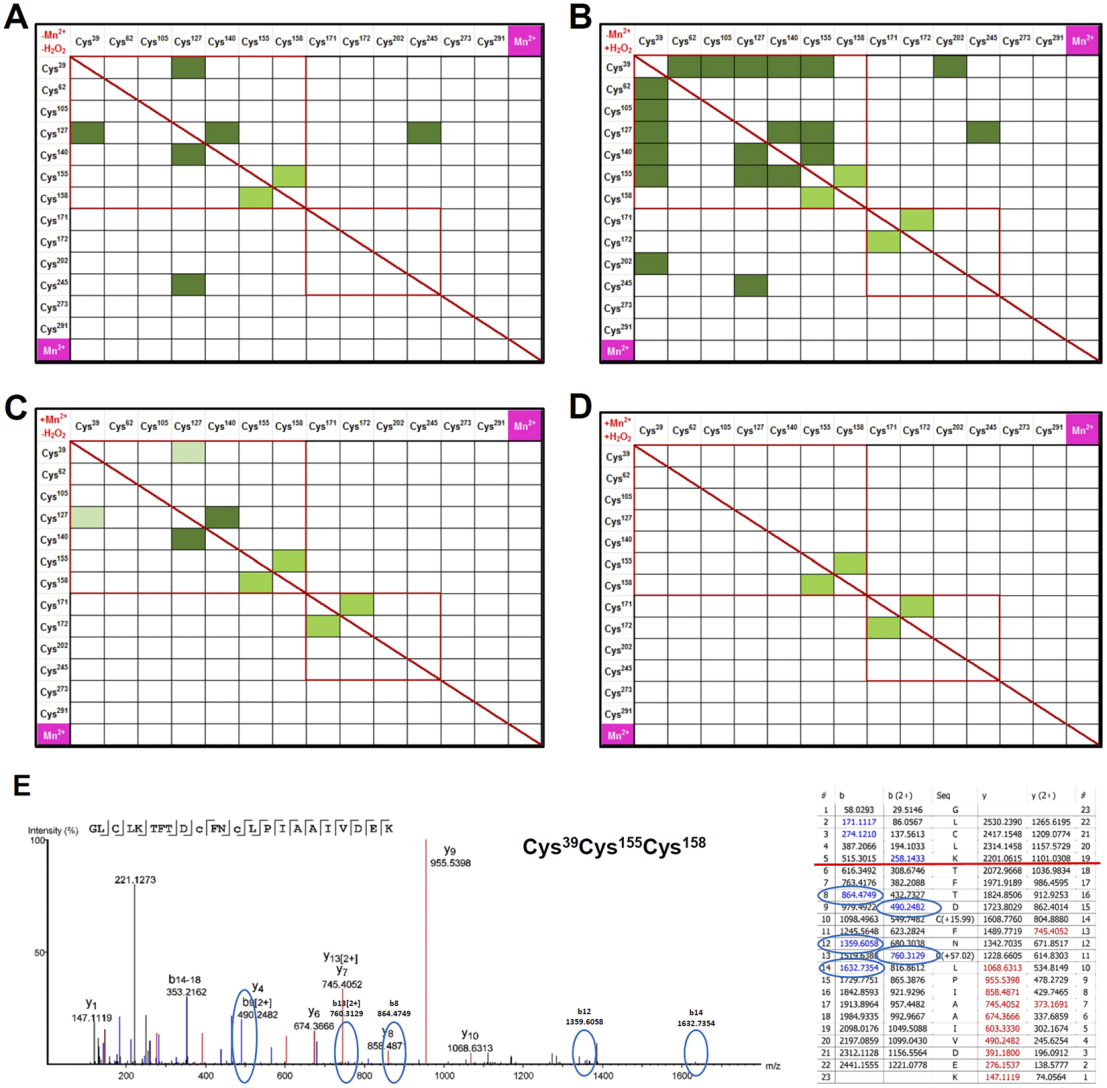
Disulfide formations in rPP-1α under different buffer conditions. (**A–D**) Cross-reactivity schemes for H_2_O_2_-depended formation of mixed (dark green) and one-peptide (light green) disulfides for GST-tagged rPP-1α. Pale green combinations are weak MS/MS spectra. The four buffer conditions with or without Mn^2+^ (100 µM) and with or without H_2_O_2_ (500 µM) were compared. (**E**) Sole disulfide spectrum (left) and ion table (right) of the Cys^39^ and Cys^155^Cys^158^ peptide identified for His-tagged rPP-1α. Red lines indicate the end of peptide1. Blue circles highlight the low abundant, but still present indicative fragments from the b-type ion series, showing unequivocally that the two peptides are interconnected with each other. The same fragment masses are highlighted in the ion table.

We next investigated whether the formation of intra-molecular transient disulfides under oxidative stress could cause for the reversible inactivation of PP-1α. The molecular distances of all Cys-residues in non-oxidized PP-1α are summarized in the cross-reactivity scheme (Fig. S3). Only few of the cysteines can likely form disulfides based on the molecular distances (within 6 Å with small structural changes). We wondered if additional S-glutathionylation is required as an intermediate stage for the formation of transient disulfide bridges. To test this, we compared disulfide formations in GST-tagged rPP-1α incubated in buffer containing 10 mM GSH with His-tagged rPP-1α without GSH under four conditions: presence or absence of 500 µM H_2_O_2_ in combination with presence or absence of 100 µM Mn^2+^, respectively. Mn^2+^ was applied to decouple the oxidative stress caused by H_2_O_2_. We plotted all identified disulfides in GST-tagged rPP-1α into cross-reactivity schemes (Fig. 3A–D). The intra-molecular disulfides Cys^155^Cys^158^ and Cys^171^Cys^172^ were identified under all conditions, which were most likely formed due to their short molecular distances and upon sample preparations because of the pKa at 8.01 and 8.97 of Cys^155^ and Cys^171^, respectively. However, the treatment with H_2_O_2_ but without Mn^2+^ led to a burst of disulfide formations, involving the surface-oriented Cys^39^ and Cys^127^, with the best spectra for Cys^127^Cys^140^ (Fig. 3B, Spectra in Fig. S6A,B).

In contrast to GST-tagged rPP-1α, the only significant disulfide formation in His-tagged rPP-1α was identified between Cys^39^ and Cys^155^Cys^158^ in the presence of H_2_O_2_ but absence of Mn^2+^ (Fig. 3E). Apparently, the molecular distance between Cys^39^ and Cys^155^ with 4.54 Å, the shortest distance among all cysteines (Fig. S3), enables this fast reaction upon H_2_O_2_ treatment. Altogether, these data suggest that additional S-glutathionylation might induce the formation of transient disulfide bridges in PP-1α.

### Quantitative approach to understand the influence of the H_2_O_2_ treatment

**Figure 4:**
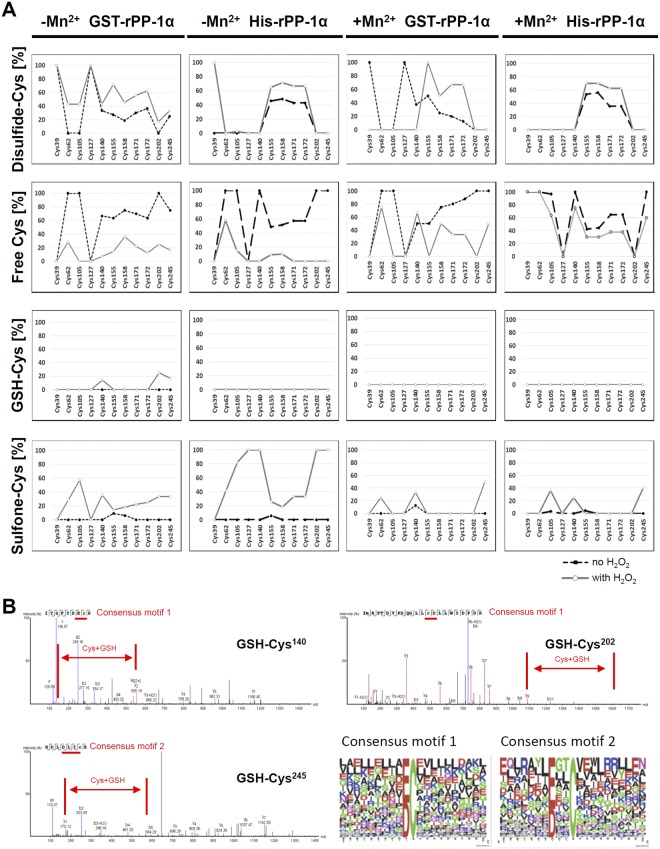
Quantitative analyses of different cysteine pools found in GST- and His-tagged rPP-1α under different conditions. (**A**) Spectral counts of disulfide-, free, GSH- and sulfone-Cys in GST-tagged and His-tagged r-PP-1α. Black dashed lines show the H_2_O_2_ treated samples and the grey solid lines the non-treated samples. (**B**) Spectra of three S-glutathionylation sites detected and the two consensus motifs they match to. Cys + GSH areas are highlighted in red.

To quantify the effect of oxidative stress on cysteine modifications in GST-tagged and His-tagged rPP-1α, we performed spectral count of free, disulfide-linked, sulfonated and glutathionylated cysteines (Fig. 4A, Table S1) and also applied LFQ quantification for the most abundant peptides (Table S2). A much-simplified quantitative response was observed for the His-tagged rPP-1α (Table S2B) compared to GST-tagged rPP-1α (Table S2A). Since Cys^273^ and Cys^291^ lie on a huge trypsin peptide, they might escape from MS detection, which can explain why we could not detect Cys^273^ and Cys^291^ in both GST-tagged and His-tagged rPP-1α. Mn^2+^ ions have a general protective effect against oxidative stress for both GST-tagged and His-tagged rPP-1α.

As mentioned above, we observed disulfide-linked Cys^155^, Cys^158^, Cys^171^ and Cys^172^ in both GST- and His-tagged rPP-1α independent of the presence of H_2_O_2_ and Mn^2+^. However, the amount of disulfide-linked Cys^155^, Cys^158^, Cys^171^ and Cys^172^ was much higher in both GST- and His-tagged rPP-1α treated with H_2_O_2_ than in their corresponding non-treated rPP-1α. Furthermore, significant amount of disulfide-linked Cys^39^, Cys^62^, Cys^105^, Cys^127^, Cys^140^, Cys^202^ and Cys^245^ was induced in GST-tagged rPP-1α upon oxidative stress in the absence of Mn^2+^ whereas only disulfide-linked Cys^39^ was induced in His-tagged rPP-1α (Fig. 4A, Table S1). These data indicate that the intra-molecular disulfide formations in GST-tagged rPP-1α upon oxidative stress depend on the presence of GSH. In GST-tagged rPP-1α, Cys^39^ and Cys^127^ were completely protected independent of H_2_O_2_ and Mn^2+^. In His-tagged rPP-1α, free Cys^39^ was observed when Mn^2+^ was included to the buffer, which was never detected in the absence of Mn^2+^ (Fig. 4A, Table S1). Cys^127^, however, was never observed as a free cysteine in both GST- and His-tagged rPP-1α, showing that Cys^39^ and Cys^127^ behave completely different in the network.

Interestingly, three cysteines Cys^202^, Cys^245^ and Cys^140^ in GST-tagged rPP-1α had an outstanding response to the H_2_O_2_ treatment: S-glutathionylation (Fig. 4B, Table S1 and S2A). A web-based glutathionylation database proposed Cys^140^, Cys^158^ and Cys^245^ in PP-1α (PDB *3N5U*) as S-glutathionylation sites^23^. Cys^202^ is a hitherto unexpected site for GSH modification. Consensus motifs for GSH sites showed acidic Asp/Glu residues in close proximity to the reactive cysteine (Fig. 4B). We also observed sulfone formation for these three cysteines in GST-tagged rPP-1α upon oxidative stress (Table S2A). Their dual response with GSH and sulfone probably reflects the solvent accessibility of Cys^140^, Cys^202^ and Cys^245^. In addition, there were more free cysteines for these three cysteines in GST-tagged than in His-tagged rPP-1α upon oxidative stress. In contrast, compared to GST-tagged rPP-1α, His-tagged rPP-1α upon oxidative stress revealed higher amount of sulfone formation of all cysteines except Cys^39^, Cys^155^, Cys^158^, Cys^171^ and Cys^172^, which fits quite well with overall less internal disulfide formation in His-tagged rPP-1α (Fig. 4A). These data indicate that significant amount of cysteines in GST-tagged rPP-1α are involved in the reversible disulfide formation and S-glutathionylation upon oxidative stress whereas more irreversible sulfonated cysteines are formed in His-tagged rPP-1α.

### Oxidations of His and Tyr in the catalytic core of rPP-1α

**Figure 5:**
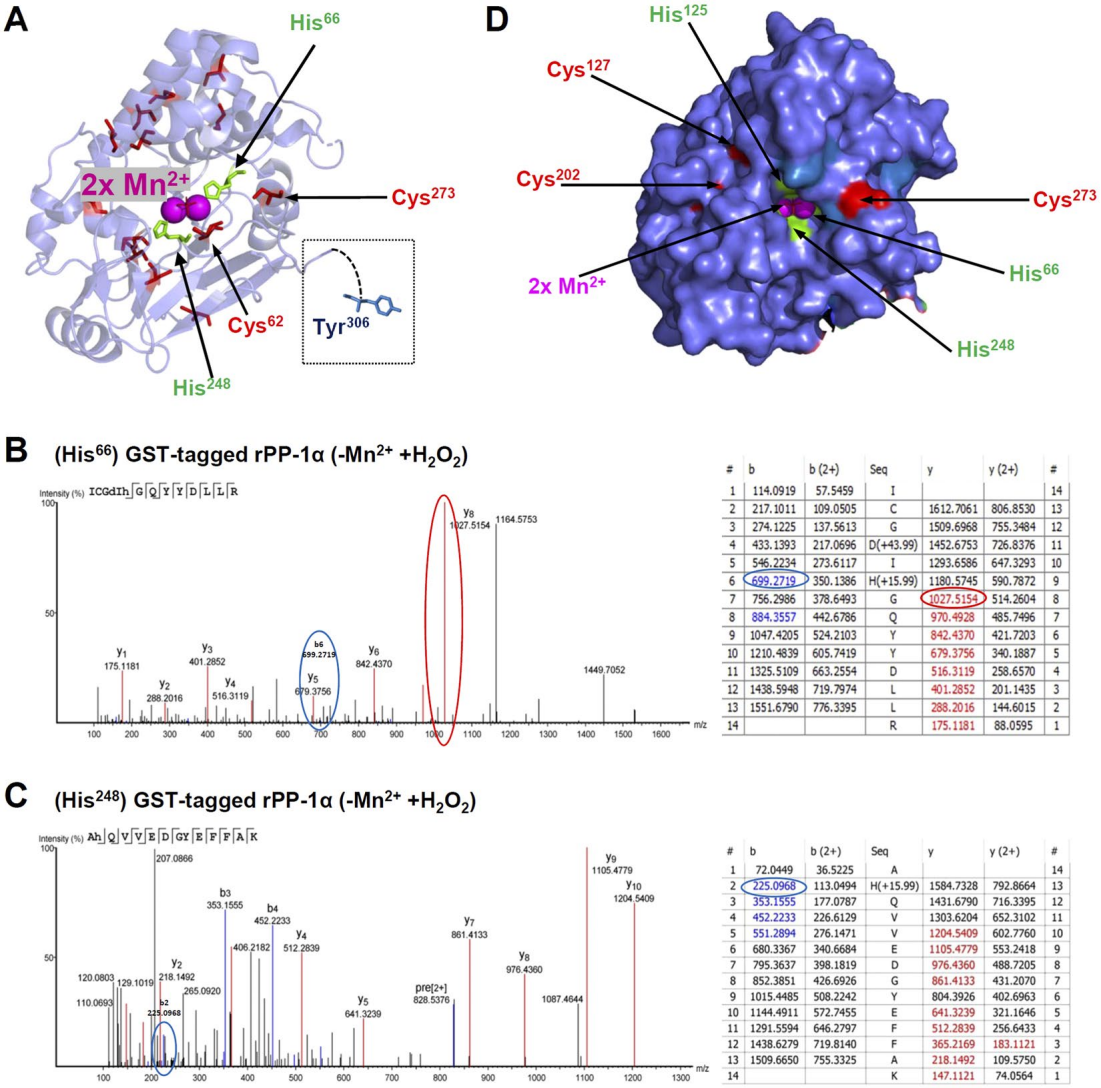
Oxidation of histidine and tyrosine residues under H_2_O_2_ treatment in GST-tagged PP-1α. (**A**) Cartoon view of oxidatively affected His^66^, His^248^ and Tyr^306^ in PP-1α. (**B-C**) Spectra and ion tables of His^66^ (**B**) and His^248^ (**C**) as indicated in blue (b-ions) or red (y-ions). (**D**) Surface view of all His- and Tyr-residues.

To further decipher the effect of oxidative stress on the activity and reaction of PP-1α, we performed additional search for the modification of other amino acids. Interestingly, out of 13 Tyr-residues, only Tyr^306^ at C-terminus of PP-1α was prone to oxidation. In His-tagged rPP-1α, we observed mono-oxidation (+15.99 Da) of Tyr^306^ without H_2_O_2_ and Mn^2+^ treatment (Fig. S7A), and di-oxidation (+31.99 Da) upon H_2_O_2_ treatment (Fig. S7B). However, in GST-tagged rPP-1α, no oxidation of Tyr^306^ was observed in the absence of Mn^2+^. When adding Mn^2+^ to the buffer, we observed unusual oxidations: +44.99 Da (~ + 15.99 Da*3–3.03 Da) and +30.01 Da (~ +15.99 Da*2–2.02 Da) of Tyr^306^ in GST-tagged rPP-1α with or without H_2_O_2_ treatment, respectively, which could result from the loss of one hydrogen/proton for each transferred oxygen (Fig. S7C,D). Although Tyr^306^ is the sole tyrosine not covered in the PDB structure (Fig. 5A), we can deduce that it must have a particularly exposed position.

An entirely different effect was observed for two out of the four His-residues, His^66^ and His^248^, in GST-tagged rPP-1α, which got mono-oxidized (+15.99 Da) under H_2_O_2_ treatment (Fig. 5B,C). They were not affected in His-tagged rPP-1α by H_2_O_2_ treatment. The X-ray structure of PP-1α strongly suggests that only His^66^, His^125^ and His^173^ are caging the two Mn^2+^ ions and that His^248^ is oriented away from the Mn^2+^ ions (Fig. 5D). Our data indicate that oxidation of His^66^ and His^248^ is shielding oxidative stress from the two Mn^2+^ ions, or, in return, transferring protons in a controlled manner, thereby controlling the oxidation state of Mn^2+^ or Mn^3+^. However, we only observed this effect in the presence of GST-activity and this opens the question if the GST-activity has an influence on the activity of PP-1α catalytic core.

### Transient disulfides as a general mechanism of proteins escaping from denaturing

To further confirm our hypothesis that formation of transient disulfide bridges might be a general mechanism of proteins to prevent from over-oxidation and irreversible inactivation, we performed the search for other redox-regulated proteins, for example, PKM2 (Fig. S8A). The 3D structure does not show any structurally relevant disulfides although there are cysteines with distances around 10 Å (Fig. S8B). We used PKM2 purified from rabbit muscle and applied H_2_O_2_ at increasing concentrations (0, 1, 10, 100, 1000 µM). We detected increasing amounts of potential disulfide precursors in PKM2 (Fig. S8C), represented by the blue dots in the heat-maps (Fig. S8D). These disulfide formations were verified by manual inspection of the spectra (Fig. S8E). Notably, these Cys-combinations are with molecular distances (>20 Å) unlikely for intra-molecular disulfides, indicating that they must be formed between two copies of a protein. However, we could not detect the dimerization in non-denaturing gels. These data suggest that non-specific dimerization of PKM2 upon oxidative stress involving Cys^49^, Cys^317^, Cys^326^ and Cys^358^ and the other cysteines at the outer domains (Fig. S8A) might protect PKM2 from denaturing.

## Discussion

In this study, we first set up the robust search strategy “cross-over-read” for MS/MS-spectra using permutated databases from non-reduced proteomics samples. Applying this method, we show that PP-1α was sensitive to H_2_O_2_ treatment and unexpected transient disulfides were formed. We demonstrate that the GST-activity was essentially required for a fast transient disulfide formation in PP-1α. The dimerization of PP-1α involving Cys^39^ and Cys^127^ is presumably important for the protection of PP-1α active surface in the absence of a substrate. Moreover, we give insight into the potential electron transport around the PP-1α catalytic core. Our data indicate that the reversible inactivation of PP-1α upon oxidative stress is, at least partially, due to transient disulfide formation as an escape mechanism against irreversible Cys-oxidation.

Although the strategy using permutated databases was used in the context of sumoylated^24^ and chemically crosslinked peptides^25^, we established here for the first time a simple approach that allowed the search of single protein entries for every disulfide-linked peptide without any enzymatic cleavage specificity. We can identify the large-molecular weight fragments, which are disconnected from the low-molecular weight fragments by a gap and normally escape from other database search algorithms, and detect both intra- and inter-molecular disulfide bridges. The PEAKS-based approach requires only MS^2^ data generated on Orbitrap XL^TM^ or QExactive^TM^. The limitation of our approach is that PEAKS identifies the spectra based on either the b-type or the y-type ions and cannot overlap both series into one spectrum. A future task would be to simplify the search workflow by overlap of the two spectrum assignments.

By applying the “cross-over-read” workflow, we identified intra- and inter-molecular transient disulfide formations in SOD1 and PKM2. The intra-molecular Cys^56^Cys^145^ in bovine SOD1 is essentially required for protein stability, correct folding and metal ion binding^22^. Two homo-di-peptides Cys^7^Cys^7^ and Cys^145^Cys^145^ identified in bovine SOD1 might be formed upon partial de-folding, which is the first step in complete denaturing and plaque formation of SOD1. In human SOD1, disulfide rearrangement was initiated with the attack of Cys^146^ rather than Cys^57^ during the intermediate state of unfolding^26,27^. PKM2 is known to exist in the catalytically distinct tetrameric and dimeric states. The dimerization of PKM2 involved transient disulfide formations upon high oxidative stress, which might be presumably important for the inhibition of PKM2 activity and to resist oxidative stress^28^. Increases in cellular ROS resulted in the inhibition of PKM2 activity through oxidation of Cys^358^, allowing the cells to withstand ROS^29^.

It is generally accepted that H_2_O_2_ serves as a signalling molecule and that its bioavailability changes modulate physiological signalling by altering the oxidation states of selected target proteins, including the kinases such as PKA, PKG and CaMKII (for details, please see the review^30^). However, little is known on the oxidative state of PP-1α^11,12^. Oxidative stress is routinely studied by exposing model systems to exogenous H_2_O_2_ with 10–1000 μM being directly relevant to biology and required to induce measureable protein oxidation or functional effects reliably^30,31^. Although peroxynitrite and hypochlorite, being at least 100- to 10000-fold more reactive towards thiols than H_2_O_2_^32^, could oxidize PP-1α at much lower concentrations in the micromolar (maybe even nanomolar) range, in our study, we did not use them due to additional effects of the reagents as nitration by peroxynitrite-derived radicals or chlorination of molecules. We show that the PP-1α activity is reduced upon H_2_O_2_ treatment. In NRCMs, H_2_O_2_ treatment resulted in phosphorylation changes of cardiac proteins such as PLB, RyR2, and cMyBPC, which are targets of PKA and PP-1α^3,33^. We also show an effect of H_2_O_2_ on I-1 phosphorylation, which is a PP-1 inhibitor and is activated by PKA^34^. H_2_O_2_ should theoretically induce phosphorylation of both PLB and cMyBPC due to the activation of PKA and inhibition of PP-1α as summarized in Fig. 1Bi. The discrepancy between PLB and cMyBPC phosphorylation in NRCMs treated with H_2_O_2_ might be speculated/explained by differences of temporal dynamics and localizations of PP-1α and PKA under oxidative stress. Since there is not much known how PP-1α and PKA activities are temporally changed at different cellular compartments under oxidative stress, we might only speculate about the time courses and localizations of PKA stimulation and PP-1α inhibition under H_2_O_2_ treatment would be different. We might speculate that PKA activity is decreased after 10 min under 100 μM H_2_O_2_ and phosphorylation of both PLB and cMyBPC is reduced. In addition, the PLB-dephosphorylation by PP-1α might be dominant after 10 min although the PP-1α activity is decreased under 100 μM H_2_O_2_. Furthermore, we detected the dimerization of PP-1α in a non-denaturing gel even without oxidative stress. Previous studies showed that the binding surface of PP-1α towards several substrates (spinophilin^21^, MYPT1^35^, NIPP1, PNUTS, RepoMAN^36^ and taperin^37^) involved Cys^202^, Cys^127^ and Cys^273^. It is very likely that dimerization between Cys^39^ and Cys^127^ directly protect the active surface of PP-1α when no substrate is around.

**Figure 6:**
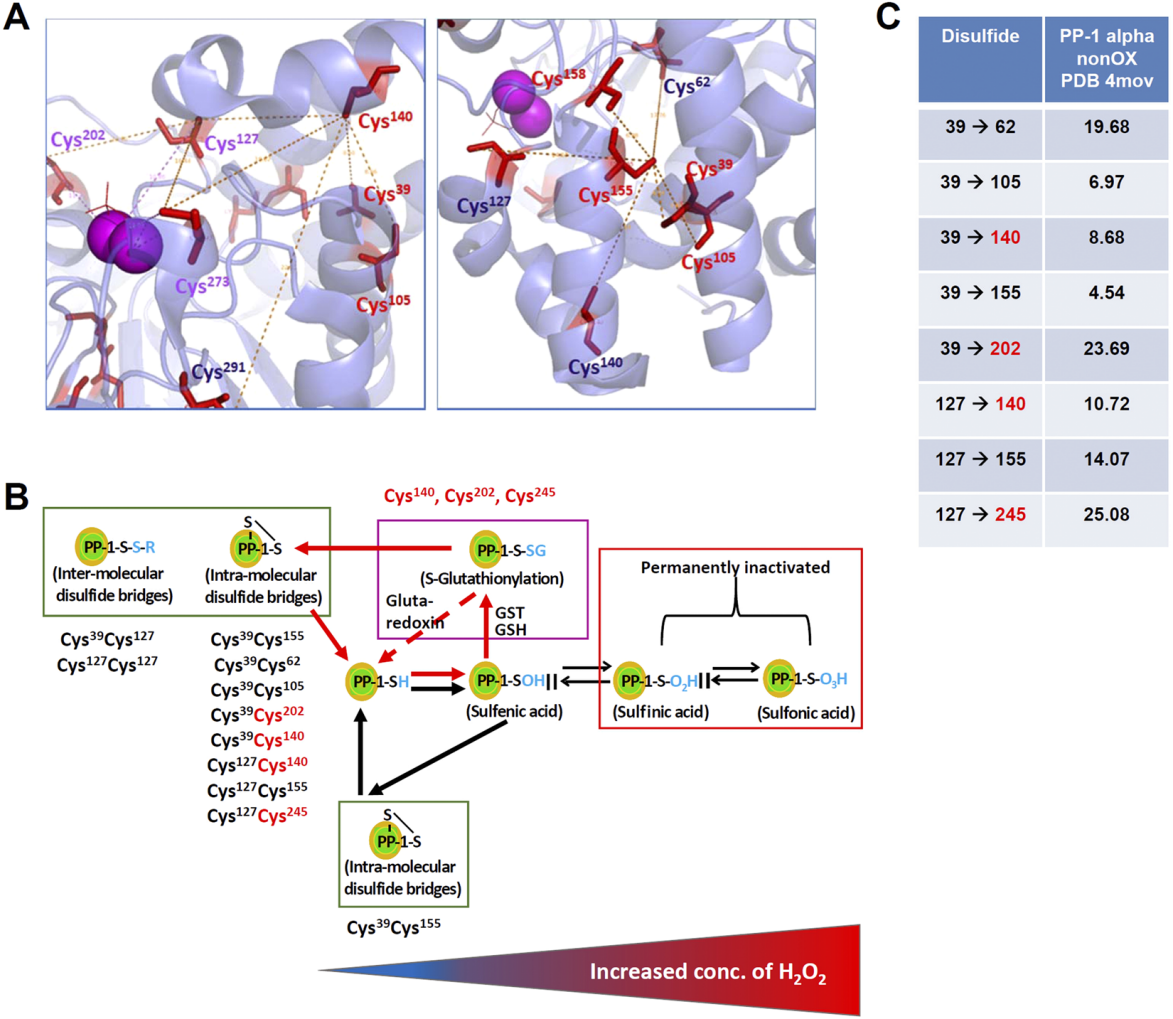
The formation of Cys^140^, Cys^202^ and Cys^245^-centered disulfide networks in PP-1α upon oxidative stress is proposed. (**A**) Cys^140^/Cys^245^/Cys^202^-centered cysteine-networks (up to 14 Å) being S-glutathionylation targets. (**B**) Connection of S-glutathionylation and formation of transient disulfides in rPP-1α (red arrows). (**C**) Summary of distances (in Å) of identified disulfides.

Most importantly, we observed the direct correlation of disulfide formations in PP-1α with increasing concentrations of H_2_O_2_, indicating that disulfides were not formed by chance or disulfide scrambling. Formation of internal transient disulfides in PP-1α under oxidative stress involved mainly either Cys^39^, Cys^127^ or one of the three glutathionylated Cys-residues Cys^140^, Cys^202^ and Cys^245^. Cys^39^, being located at the surface of the PP-1α protein and together with Cys^155^ and Cys^158^ being defined as the catalytic centre of PP-1α, might be both the activity regulator and the backdoor cysteine (Fig. 6A). Under harsh oxidative conditions, Cys^39^ is not available anymore because it serves as a backdoor cysteine to protect Cys^155^ and Cys^158^, therefore, we identified only the homo-di-peptide Cys^127^Cys^127^. Moreover, many mixed disulfides involving Cys^39^ and Cys^127^ are believed to freeze conformational deformation of PP-1α. In general, the advantages of this structural modification would be that misfolding states are required before the correct folding is established^38^, proteins obtain more thermostability^39^ and over-oxidation of catalytically active cysteines is prevented^40^. An oxidoreductase active site, which is highly conserved within the PP-1 subfamily, but not in the PP-2A or -2B subfamilies, was identified in close proximity to the phosphatase active site, suggesting a regulatory control mechanism^41^. Since the active site of PP-1α (Cys^155^, Cys^158^, Pro^192^) is protected by Cy^39^, we might speculate that the dimerization of PP-1α via Cys^39^ is actively controlled and might explain the detection of PP-1α dimerization under all conditions tested.

Notably, we detected S-glutathionylation of Cys^140^, Cys^202^ and Cys^245^ in GST-tagged rPP-1α upon high oxidative stress. This is in line with previous studies showing that S-glutathionylation occurs through the reversible addition of glutathione to thiolate anions of cysteines in the presence of GST. This modification serves both to protect and to modify structure/function^42^. Previous study showed that both PP-1 and PP-2A activity was inhibited by different oxidizing agents including oxidized glutathione (GSSG) though the kinetics of inactivation of two enzymes were different^43^. Interestingly, the addition of GSH could reactivate both enzymes, which might involve Cys^140^ of PP-1^43^. Cys^140^ is found as an essential glutathionylation site in our study. It was also reported that PP-2A activity was inhibited in Caco-2 cells treated with 20 µM H_2_O_2_ or GSSG and the inhibition was restored by GSH^44^. Therefore, we present here the Cys^140^, Cys^202^ and Cys^245^-centered disulfide networks (Fig. 6A) and propose that S-glutathionylation is required as a fast response to oxidative stress by forming mixed disulfide bridges, explaining how PP-1α functions as a redox sensor (Fig. 6B). Cysteines with distances even >20 Å (Fig. 6C) might be bridged upon conformational changes when the additional fast reaction with GSH activates the cysteines for disulfide formation. Moreover, other PTMs might be also involved in the redox regulation of enzyme activities, for example, reversible S-nitrosation of thiolate-containing enzymes involved in providing reducing equivalents is considered a redox-regulated mechanism to keep mitochondria in a protected oxidized state under conditions when an excess of nitric oxide was generated, ultimately leading to the formation of the nitrosating species N_2_O_3_^45^. By this way, components of the respiratory chain are reversibly maintained in an oxidized state to transiently protect these complexes from an irreversible modification. We may speculate that both mechanisms (formation of transient disulfides and S-nitrosation) might occur in the cell depending on the subcellular location and the availability of nitrite.

Recently the formation of transient disulfides within KIM-like tyrosine phosphatases is also linked with the reversible inactivation of the phosphatase activity^46^. Compared to KIM-like tyrosine phosphatase, PP-1α is different in many aspects, for instance, the dimerization is not oxidative stress dependent, but would rather control the surface availability for binding of substrate proteins. Our data demonstrate that PP-1α is more robust to withstand higher levels of redox stress − 50% inactivation is observed at 500 µM H_2_O_2_ for PP-1α, but already at 150 µM H_2_O_2_ for the KIM-like tyrosine phosphatases. However, it is largely unknown how oxidative stress-induced disulfide formations in PP-1α could influence its interaction with other proteins and the substrate binding. Previous study showed that oxidative stress led to formation of stable complexes containing PP-1α, GADD34, elF2a and TDP-43 and enhancing the substrate binding^47^.

Furthermore, we propose a mechanism how the electron current is established in/out the catalytic centre of PP-1α. Solely, Cys^273^ is only 4.4 Å away from Mn^2+^ (Fig. 6A), 3.68 Å from His^66^ and 8.55 Å from His^248^ and all these residues are located at the entry to the cavity (Fig. 5D). It is likely that GST-mediated reduction of Cys^273^ can organize an electron flow in/out of the cavity. Moreover, we found several oxidation states of His^248^ and His^66^ and oxidation of Tyr^306^. It is known that tyrosines and histidines are prone to oxidation and might also co-act as an electron acceptor/transfer pair^48^. Histidines alone can play a role in the oxidation of metal ions^49^. It was shown that PP-1 activity mainly relied on the di-nuclear metal centre, rather than Cys-redox modifications for catalysis^50,51^. Recently, elevated NOX2 activity in the mouse heart resulted in PP-1α inactivation that involves metal centre oxidation rather than the thiol oxidation^11,12^. Altering the redox state of Mn^2+^-Mn^2+^ to Mn^3+^-Mn^2+^ or Mn^3+^-Mn^3+^ can shorten the bond lengths between the metal ions and the ligands and increase the energy barrier of the related reactions^52^.

One limitation of our study is that we investigated the effect of H_2_O_2_ on Cys-modifications of PP-1α using recombinant proteins. Cys-modifications of PP-1α might be different in the heart under oxidative stress. Future study should be conducted in cells or tissues. Another limitation is that we studied only some oxidative modifications of protein thiols. Besides intra- and inter-protein disulfides, S-glutathionylation and S-sulfonation we have studied, protein thiols can form other oxidative modifications, including reversible (S-sulfenation, S-nitrosation, S-sulfhydration, S-sulfenamidation) and irreversible (S-sulfination) redox states. To decipher the mechanism how redox stress regulates PP-1α activity, further studies have to be performed to figure out under which conditions irreversible modifications S-sulfination or S-sulfonation occur, which Cys-residues are involved and how intermediate states such as S-sulfenation, S-nitrosation or S-sulfhydration transition to disulfides^53^.

In conclusion, our study demonstrates that the formation of transient disulfides in PP-1α is involved in sheltering against oxidative stress. For a fast response, GST-activity is required. Thus, the GSH-mediated formation of transient disulfides might help proteins to escape from irreversible cysteine oxidation and to prevent their complete inactivation (Fig. 6B). These data suggest that protein S-glutathionylation might act as a valuable biomarker for oxidative stress, with potential for translation into novel therapeutic strategies^54^.

## Material and Methods

### Animals

All animal experiments were performed in accordance with the guidelines from Directive 2010/63/EU of the European Parliament on the protection of animals used for scientific purposes. All procedures involving animals were approved by the Niedersächsisches Landesamt für Verbraucherschutz und Lebensmittelsicherheit (Germany).

### Live-cell imaging of NRCMs

NRCMs were isolated at postnatal day 1–3 and cultured in a 6-well plate for 3–4 days. 90 min before live-cell imaging, NRCMs were incubated with 0.1, 1 or 10 mM H_2_O_2_ in the climate chamber of the Olympus fluorescence microscope (37 °C, 5% CO_2_). The video frames were recorded with 57.37% lamp intensity every 20 min with an exposure time of 20 ms for 24 h. A matlab based script was used to generate heat maps of the mean contraction for each given H_2_O_2_ concentrations^18^. A resolution of 0.6442 μm/pixel was used and dead floating cells were filtered. The changes in the morphology of NRCMs were analysed with Image J.

### Phosphatase activity assay

Total phosphatase activity was measured using the EnzChek Kit (Molecular Probes) as previously described^55^. NRCMs were harvested in a passive lysis buffer (20 mM Tris-HCl pH 7.5, 1 mM Na_2_EDTA, 150 mM NaCl, 1 mM EGTA, 1% Triton and complete protease inhibitor (Roche)). The protein content was measured using Pierce BCA Protein Assay Kit. To analyze phosphatase activity, 6,8-difluoro-4-methylumbelliferyl phosphate (DiFMUP) was used as a substrate to mimic phosphorylated proteins, which does not fluoresce. Upon dephosphorylation by phosphatase, DiFMUP changes to DiFMU and becomes highly fluorescent. 100 µM DiFMUP as substrate was pre-mixed with 100 mM sodium acetate (reaction buffer, pH 5.5), and then incubated with 20 µg total protein at RT for 15 min. Fluorescence values of converted DiFMU were read on a Flexstation 3 (Molecular Devices). Linearity of the assay as a function of PPase activity was tested using standards of commercially available recombinant PP-1 (Sigma Aldrich, SRP5338).

*In-vitro* phosphatase activity assay (Promega) for rPP-1α was performed in 96-well format according to the manufacturer’s introduction: (i) reaction of rPP-1α in the buffer containing MgCl_2_ and MnCl_2_ using non-fluorescent phosphorylated bisamide rhodamine 110 (R110) and 7-amino-4-methylcoumarin (AMC) as substrates; (ii) addition of H_2_O_2_ at different concentrations; (iii) stopping reaction after 10 min by adding protease; (iv) digestion of R110 and AMC to generate highly fluorescent R110 and AMC; and (v) calculation of the R110/AMC ratio as a measurement of the PP-1α activity.

### SDS-PAGE and quantification

After incubation with H_2_O_2_, NRCMs were lysed with ice-cold GST-fish buffer (10% glycerol, 50 mM Tris pH 7.4, 150 mM NaCl, 1% NP-4, 4 mM MgCl_2_, 1% IGEPAL CA-630, complete protease inhibitor), cell debris was removed at 13,000 *g* (10 min, 4 °C). For non-reducing 12–15% acrylamide SDS-PAGE, the cells were lysed in the presence of 10 mM maleimide but without DTT and heat. Gels were blotted onto a nitrocellulose membrane, blocked with 1x Roti-Block (Roth) for 1 h, washed with TBST (Tris-buffered saline, 0.1% Tween 20) and incubated overnight at 4 °C with a primary antibody: calsequestrin (1:1000, Source: rabbit, Dianova, ABR-01164), PP-1α (1:200, Source: goat, Santa-Cruz, sc-6104), PP-2Ac (1:1000, Source: rabbit, Millipore, 07–324), PKA-RI (1:200, Source: mouse, BD Transduction laboratories, 610166), PLB-pSer^16^ (1:5000, Source: rabbit, Badrilla Ltd., A010–12), cMyBPC-pSer^282^ (1:5000, Source: rabbit, Enzo Life Science, ALX-215–057-R050), and I-1-pThr^35^ (1:1000, Source: rabbit, Cell signalling, #2302). The membrane was washed three times with TBST for 10 min each and incubated for 1 h with the secondary antibody: HRP-coupled antibodies (mouse: Sigma-Aldrich, A3682; rabbit: Sigma-Aldrich, A0545; goat: Santa-Cruz, sc-2020).

### Oxidative stress experiments on rPP-1α and PKM2

100 ng GST-tagged rPP-1α (Sigma-Aldrich SRP5338), supplied in 50 mM Tris-HCl, pH 7.5, 150 mM NaCl, 10 mM glutathione, 0.1 mM EDTA, 0.25 mM DTT, 0.1 mM PMSF, and 25% glycerol, was incubated for 15 min under four conditions: (1) -Mn^2+^, -H_2_O_2_, (2) -Mn^2+^, 500 µM H_2_O_2_, (3) 100 µM Mn^2+^, -H_2_O_2_, (4) 100 µM Mn^2+^, 500 µM H_2_O_2_. 10 mM iodoacetamide (IAA) was immediately added and samples incubated for 20 min. Experiments were repeated with 100 ng of His-tagged rPP-1α (Sigma-Aldrich P7937) or PKM2 (Sigma-Aldrich SRP0415) in the absence of glutathione.

### PyMOL analysis

PyMOL (Version 2.0.6 Schrödinger, LLC) was used to visualize protein structure and to measure molecular distances of all Cys-residues and Mn^2+^. A PyMOL script file was prepared for selection of several residues and their labelling either as coloured sticks or balls.

### LC-MS/MS

Non-treated bovine SOD1 (Sigma Aldrich S7571) or H_2_O_2_-treated recombinant proteins were mixed with 4% SDS to obtain a final SDS concentration of 1%. Thereafter, the samples were subjected to filter-aided sample preparation^56^. We performed buffer exchanges twice: (i) 8 M urea, (ii) 50 mM ammonium bicarbonate (ABC). Overnight digestion (~18 h) was performed at 37 °C in 100 µl 50 mM ABC with 1 µg trypsin/chymotrypsin. Sample was purified by spinning the filters with 50 µl 50 mM ABC, 50 µl 0.5 M NaCl and 100 µl StageA (0.1 M acetic acid) and desalting via in-house StageTips (3 discs Empore SPE Disks) and eluted with 40 µl StageB (80% (v/v) acetonitrile, StageA) two times into 96-well microtiter plates, dried and reconstituted in StageA^57^.

The Q Exactive Plus system was interfaced with a Dionex Ultimate 3000 ultra-high-performance liquid chromatography and a Nanospray Flex Ion-Source (all from Thermo Fisher Scientific). For GST-tagged rPP-1α, peptides were loaded onto one of the two C18 PepMap100 columns (300 µm × 5 mm, 5 µm particle size, 100 Å pore width) at the flow rate 25 µl/min (1% acetonitrile (v/v), 0.1% formic acid (v/v) in water). Alternative elution in 28.5 min or 31.0 min from the pre-columns and separation on an in-house column in liquid junction emitters (100 µm i.d, tip 10 ± 1 µm, New Objective) using 2.4 µm Reprosil Saphir (Dr. Maisch GmbH) was performed at 0.5 µl/min by a binary gradient of buffer B (80% acetonitrile (v/v) in 0.1% formic acid) from 5% to 44%, followed by 4 min wash-out at 99% buffer B and 10 min re-equilibration to 1% buffer B. For the His-tagged rPP-1α, separation time was 60 min with only one pre-column installed.

For GST-tagged rPP-1α, ionization was conducted at 2.6 keV using liquid junction and MS spectra were recorded with resolution of 70,000 at 200 m/z in profile mode, MS-AGC (automatic gain control) at 3 × 10^6^, maximal injection time of 250 ms, and MS-mass range of 300–2500 m/z. MS/MS precursors with z = 2–6 were selected by applying isolation window m/z = 2, intensity threshold of 6.7 × 10^3^, MS/MS-AGC target value of 10^5^, underfill ratio of 1%, and maximal injection time of 150 ms. MS/MS spectra were recorded with resolution of 17,500 at 200 m/z, higher-energy collisional dissociation (HCD) at normalized energy of 30 keV, MS/MS-mass range of 200–2000 m/z, and dynamic exclusion of 30 sec (10 ppm). For His-tagged rPP-1α, ionization was conducted at 3.3 keV using direct junction (stainless steel emitter). Changed parameters were: maximum injection time of 160 ms, MS-mass range of 200–2000 m/z, and stepped HCD at 30, 35 and 40 keV.

PKM2 was measured on the LTQ-Orbitrap (Thermo Fisher Scientific) coupled to an Easy Nano LC system 300 I (Proxeon for Bruker). Sample was loaded onto Dr. Maisch Reprosil Pur precolumn cartridge (5 µM 120 Å, ID 300 µm) in 10 min at 2 µl/min in 5% buffer B (95% acetonitrile, 0.1% formic acid). Peptides were eluted at a flow rate of 280 nl/min in-line within 60 min by a gradient of buffer B from 5% to 95%, followed by wash-out with 95% buffer B for 10 min and equilibration with 5% buffer B for 10 min. MS spectra were recorded with resolution of 60,000 for 0.3 sec. MS/MS spectra of the same target mass (1^st^ ion-trap, 2^nd^ Orbitrap) were generated upon overlap of two collisional induced dissociation (CID), and with AGC of 10^4^ ions, and fragmentation energy at 35 keV. MS/MS resolution was either “enhanced” for the ion-trap or at 7,500 for the Orbitrap.

### Data analysis with PEAKS software

Spectra were searched with PEAKS 7.0/7.5^58^. The maximum variable modifications allowed were carbamidomethylation (+57.02), Cys-oxidation or sulfone (+47.98), glutathione disulfide (+305.07), Cysteinyl C (+119.00), deamidation (NQ) (+0.98), disulfide bridge unpaired fragmentation (−2.02), dehydroalanine C (−33.99), dioxidation M (+31.99), hydroxylation (+15.99), oxidation M (+15.99), oxidation HW (+15.99), oxidation C (+15.99), persulfide C (+31.97) and phosphorylation STY (+79.97). Databases used were Uniprot human (July7^th^ 2015, 68,605 entries), rabbit (21183 entries) and bovine (Sep30^th^ 2015, 32,194 entries). Mass tolerances was 10 ppm for MS and 0.02 Da for MS/MS. The changed trypsin specificity was accepted with four missed-cleavages and cutting before proline. For the His-tagged rPP-1α, additional variable modifications were acetylation (N-terminus) (+42.01), arginine oxidation to glutamic semialdehyde (−43.05), disulfide CID breakage (+33.99), dehydration (−18.01), internal disulfide bond (−1.01), Methyl ester (+14.02) and proline oxidation to pyroglutamic acid (+13.98). Quantification of peptides was performed by spectral count in PEAKS or by LFQ in MaxQuant 1.528^59^.

For the identification of disulfide spectra, we set up permutated cysteine peptide databases in PEAKS 7.5. We did an in-silico trypsin digest of the amino acid sequences of the proteins of interest and extracted all cysteine peptides (CP = [cp_1_, …, cp_n_], |CP| = n). The sequences for the database were derived by appending the sequence of cysteine peptide2 to the sequence of cysteine peptide1 with 1, 2 ∈ CP for all possible pairs of 1 and 2 (DB = [cp_1,1_, …, cp_1,n_, cp_2,1_, …, cp_n,n_], |DB| = n^2^). Each pair was then stored within a unique entry in a FASTA-file while discarding duplicate sequences.

The fragmentation behaviour of the disulfide linked di-peptide was similar to SUMO1-modified peptides^24^. It stopped before the first Cys and continued at higher molecular weight for the other peptide. The hidden C-terminus of peptide1-peptide2 or peptide2-peptide1 was modulated by an additional PTM at K/R for trypsin and at Y/L/W/F/I/M for chymotrypsin: −2.02 a.m.u. + 18.01 a.m.u. = +15.99 Da.

## Electronic supplementary material

Supplementary Information
