# Supplementary material for: The reduced activity of PP-1α under redox stress condition is a consequence of GSH-mediated transient disulfide formation
Source: Sci Rep. 2018 Dec 7;8:17711. doi: 10.1038/s41598-018-36267-6 (PMC6286341; doi:10.1038/s41598-018-36267-6)
Supplement: Supplementary file 1 — Supplementary Information [file 41598_2018_36267_MOESM1_ESM.pdf]

Supplementary Information

The reduced activity of PP-1α under redox stress condition is a consequence of GSH-mediated transient disulfide formation

Simranjit Singh, Simon Lämmle, Heiko Giese, Susanne Kämmerer, Stefanie Meyer-Roxlau, Ezzaldin Ahmed Alfar, Hassan Dihazi, Kaomei Guan, Ali El-Armouche, Florian Richter

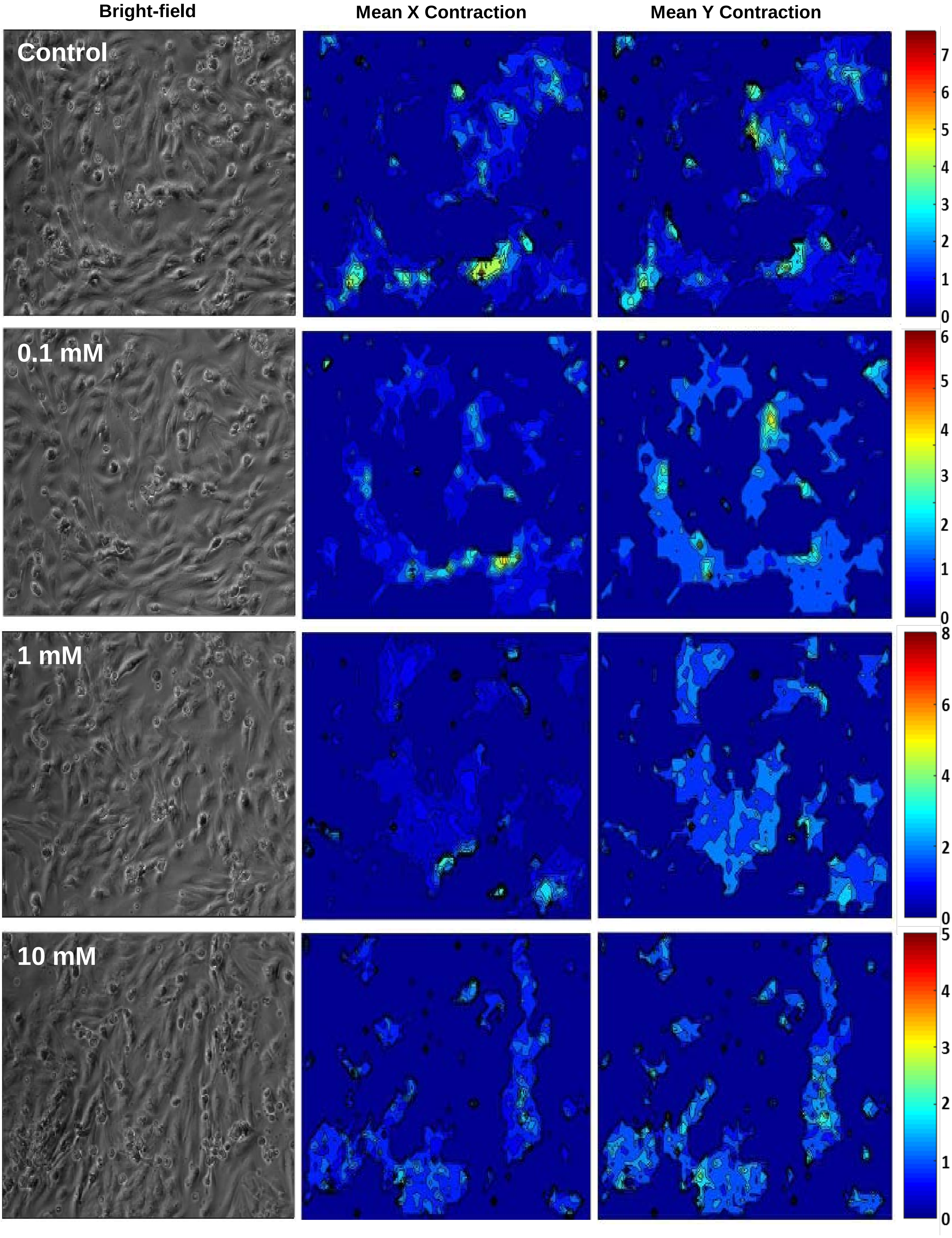

Figure S1. Original live cell images and the first processing steps that lead to the motion pictures in Figure 1A.

A

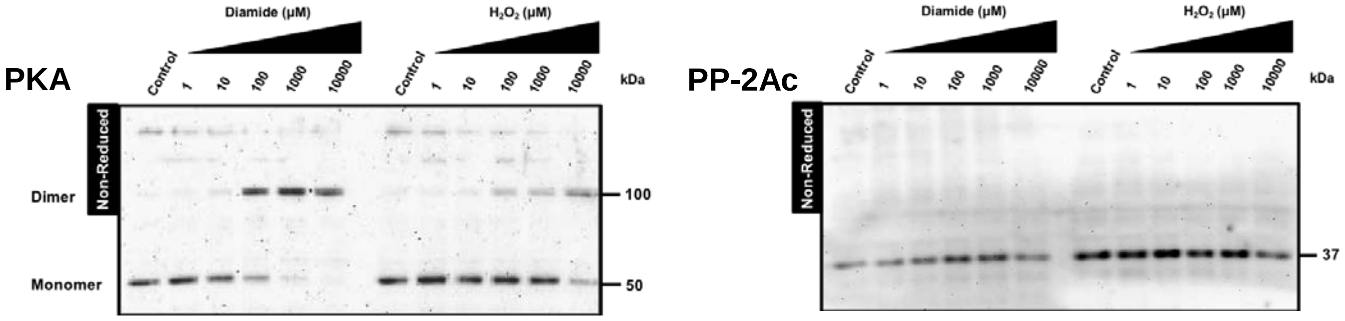

B

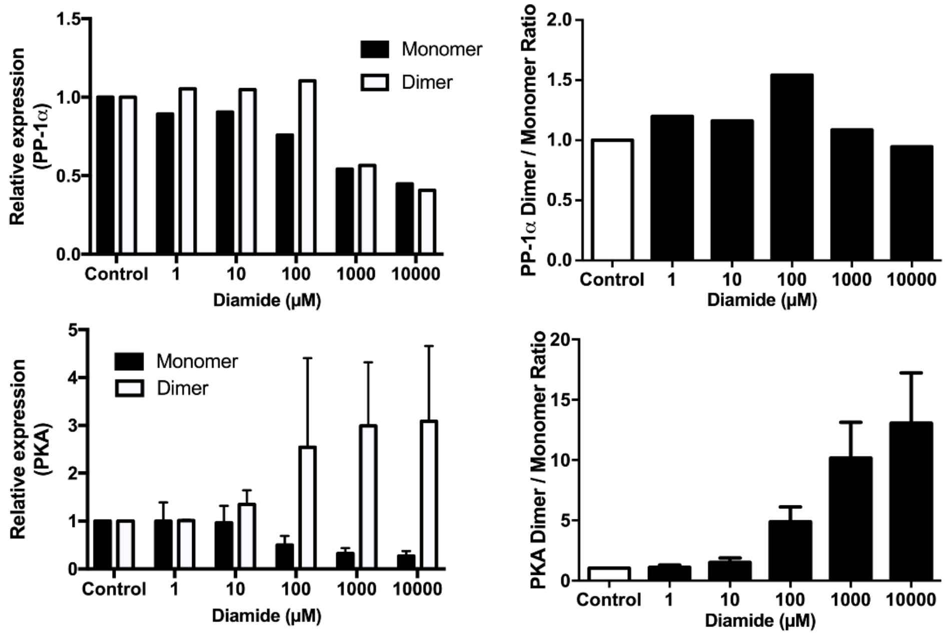

C

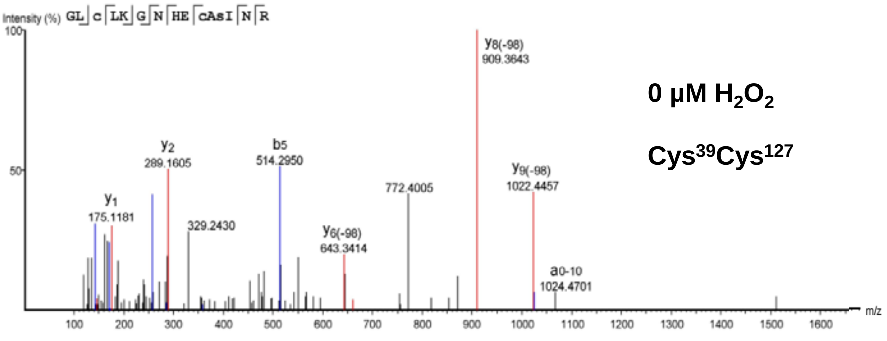

0 μM H_2O_2

Cys[39]Cys[127]

#	b	b (2+)	Seq	y	y (2+)	#
1	58.0293	29.5146	G			15
2	171.0754	86.0567	L	1634.7018	817.8509	14
3	273.1135	137.0568	C(-1.01)	1521.6177	761.3088	13
4	386.1976	193.5988	L	1419.6174	710.3087	12
5	514.2950	257.6515	K	1306.5334	653.7667	11
6	571.3140	286.1372	G	1178.4385	589.7192	10
7	685.3569	343.1785	N	1121.4170	561.2085	9
8	822.4158	411.7079	H	1007.3741	504.1870	8
9	951.4584	476.2292	E	870.3152	435.6576	7
10	1052.4496	526.7248	C(-2.02)	741.2726	371.1363	6
11	1123.4867	562.2433	A	640.2814	320.6407	5
12	1290.4851	645.7426	S(+79.97)	569.2443	285.1221	4
13	1403.5691	702.2845	I	402.2459	201.6230	3
14	1517.6121	759.3060	N	289.1605	145.0962	2
15			R	175.1181	88.0595	1

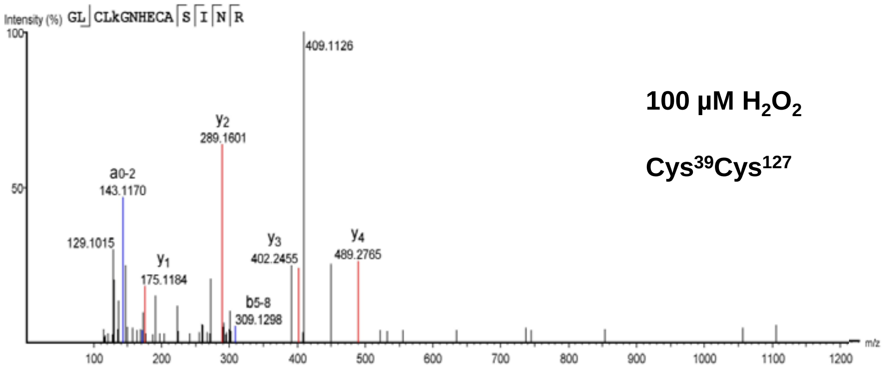

100 μM H_2O_2

Cys[39]Cys[127]

#	b	b (2+)	Seq	y	y (2+)	#
1	58.0293	29.5146	G			15
2	171.1116	86.0567	L	1573.7573	787.3787	14
3	274.1225	137.5613	C	1460.6732	730.8366	13
4	387.2066	194.1033	L	1357.6641	679.3320	12
5	531.2964	266.1482	K(+15.99)	1244.5801	622.7900	11
6	588.3179	294.6590	G	1100.4901	550.7451	10
7	702.3608	351.6804	N	1043.4686	522.2343	9
8	839.4198	420.2099	H	929.4258	465.2129	8
9	968.4623	484.7312	E	792.3668	396.6834	7
10	1071.4716	536.2358	C	663.3242	332.1621	6
11	1142.5087	571.7543	A	560.3151	280.6575	5
12	1229.5406	615.2703	S	489.2765	245.1390	4
13	1342.6248	671.8124	I	402.2455	201.6230	3
14	1456.6677	728.8339	N	289.1601	145.0809	2
15			R	175.1184	88.0595	1

D

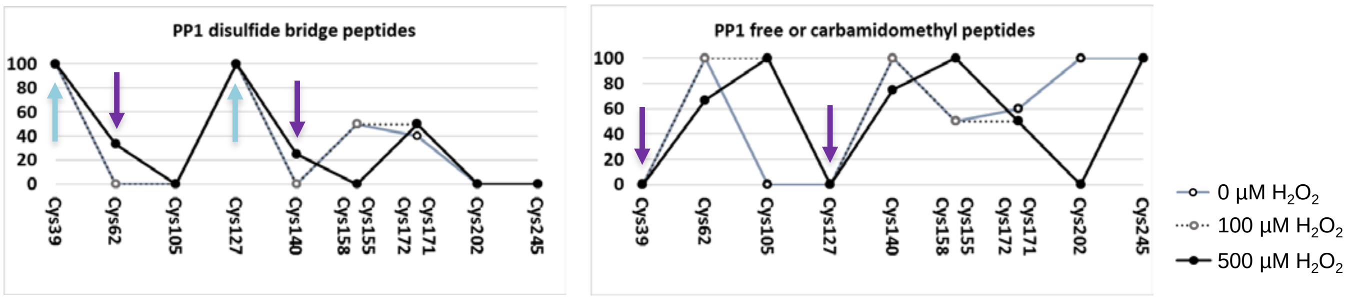

Figure S2. Detection of protein dimerization. (**A**) Dimer formation for PKA and PP-2A in NRCMs treated with increasing concentrations of diamide or H_2O_2. (**B**) Quantification of monomer and dimer form of PP-1α and the dimer to monomer ratio in case of oxidative stress-independent dimerization from Figure 2A (n=2). (**C**) Quantification of dimer to monomer form of PKA and the monomer to dimer ratio in case of oxidative stress-independent dimerization from Figure S2A (n=3). (**D**) Two $Cys^{39}Cys^{127}$ disulfide spectra found in rPP-1α with and without H_2O_2 treatment. (**E**) Quantification of free cysteine and disulfide spectra identified at increasing concentrations of H_2O_2 (0, 100, 500 µM).

	Cys^{39}	Cys^{62}	Cys^{105}	Cys^{127}	Cys^{140}	Cys^{155}	Cys^{158}	Cys^{171}	Cys^{172}	Cys^{202}	Cys^{245}	Cys^{273}	Cys^{291}	Mn^{2+}	Color code
Cys^{39}		19.68	6.97	15.72	8.68	4.54	16.54	24.20	22.15	23.69	25.46	23.86	33.78	19.92	30.00
Cys^{62}	19.68		6.82	18.44	22.31	17.76	10.41	8.60	7.78	19.53	6.91	18.04	13.11	11.82	27.00
Cys^{105}	6.97	6.82		18.96	8.66	9.65	16.64	24.81	23.01	7.04	25.08	21.18	30.20	20.19	24.00
Cys^{127}	15.72	18.44	18.96		10.72	14.07	13.94	21.41	15.82	12.56	23.80	16.44	28.94	11.00	21.00
Cys^{140}	8.68	22.31	8.66	10.72		11.64	16.65	27.56	23.75	23.16	27.53	18.62	33.79	17.45	18.00
Cys^{155}	4.54	17.76	9.65	14.07	11.64		6.98	20.33	18.59	20.82	22.60	23.76	27.07	17.75	15.00
Cys^{158}	16.54	10.41	16.64	13.94	16.65	6.98		20.98	19.30	12.02	23.67	27.99	29.58	19.72	12.00
Cys^{171}	24.20	8.60	24.81	21.41	27.56	20.33	20.98		5.96	19.42	4.57	24.22	9.08	15.24	10.00
Cys^{172}	22.15	7.78	23.01	15.82	23.75	18.59	19.30	5.96		13.56	6.43	19.11	15.03	19.24	9.00
Cys^{202}	23.69	19.53	7.04	12.56	23.16	20.82	12.02	19.42	13.56		19.87	21.33	26.59	12.41	8.00
Cys^{245}	25.46	6.91	25.08	23.80	27.53	22.60	23.67	4.57	6.43	19.87		21.25	7.15	14.37	7.00
Cys^{273}	23.86	18.04	21.18	16.44	18.62	23.76	27.99	24.22	19.11	21.33	21.25		26.60	9.08	6.00
Cys^{291}	33.78	13.11	30.20	28.94	33.79	27.07	29.58	9.08	15.03	26.59	7.15	26.60		20.86	5.00
Mn^{2+}	19.92	11.82	20.19	11.00	17.45	17.75	19.72	15.24	19.24	12.41	14.37	9.08	20.86		4.00

Figure S3. Cross-reactivity table for all cysteines and the Mn^{2+} ions in PP-1α showing the molecular distances measured in the PDB-file *4MOV*. On the right, colour code is given for the molecular distances. Two networks of possibly interacting cysteines are made up on the left upper corner and on the right lower corner, which separate the protein into two halves with potentially different accessibility to oxidative stress. On the rim, the cysteines involved in dehydroalanine or persulfide formation are shown in dark green. The cysteines involved in glutathionylation are shown in grey.

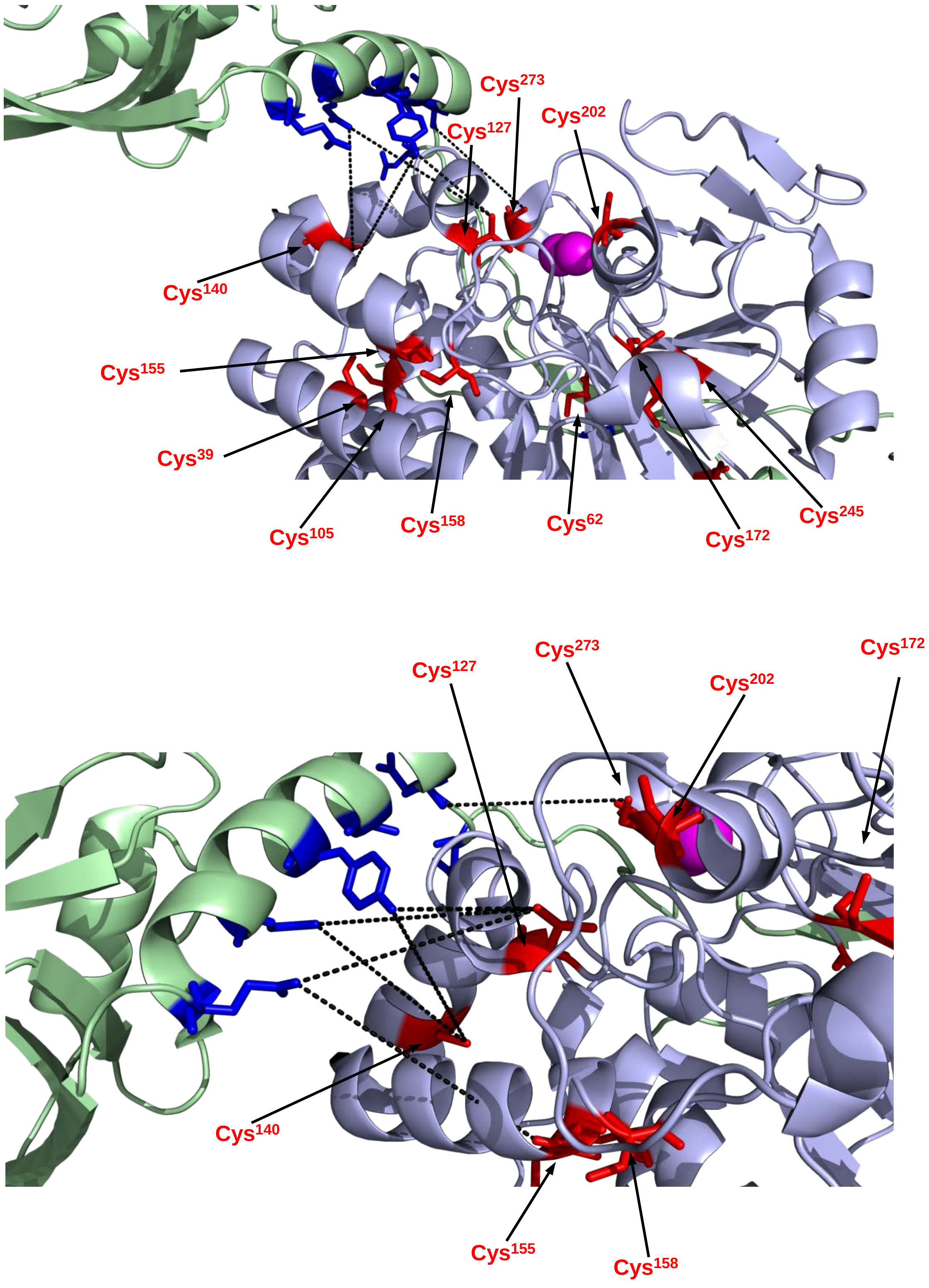

Figure S4. Two views of the interaction surfaces between PP-1α (light blue cartoon view) and spinophilin (light green cartoon view) prepared from the PDB structure *3egg*. Hydrophilic negatively charged residues of spinophilin are shown in dark blue and the cysteines of PP-1α are shown in red. Interestingly, the major substrate binding site for spinophilin includes interactions of Cys^{140}, Cys^{127}, Cys^{273} and eventually Cys^{202}, which are relevant for dimerization of PP-1α (Cys^{127}), involved in the catalytic activity (Cys^{273}) or protected by GSH being surface exposed (Cys^{140}, Cys^{202}).

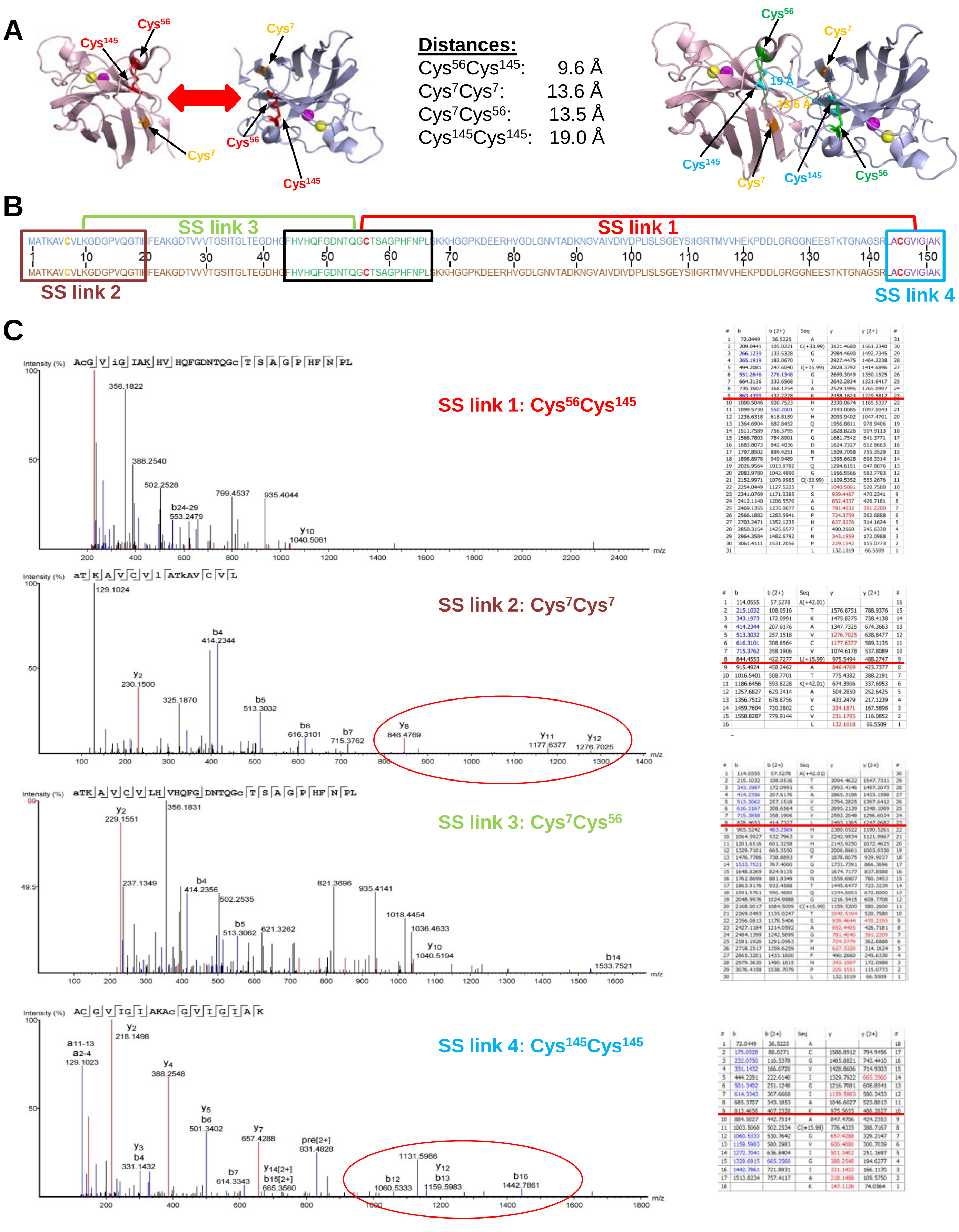

Figure S5. Validation of our MS/MS-spectrum search strategy using bovine SOD1. (**A-B**) Disulfides identified in SOD1 are shown in distinct colours on the structure (**A**) and in the sequences (**B**). SS link 1: Cys[56]Cys[145] in red, SS link 2: Cys[7]Cys[7] in brown, SS link 3: Cys[7]Cys[56] in green, and SS link 4: Cys[145]Cys[145] in blue. SS link Cys[56]Cys[56] in black was never detected. The disulfide between Cys[56] and Cys[145] (red sticks) in SOD1 is required for correct folding and metal ion binding (yellow and pink balls). (**C**) Spectra (left) and ion tables (right) of the detected disulfide peptides. B-type ions are in blue and y-type ions in red.

A

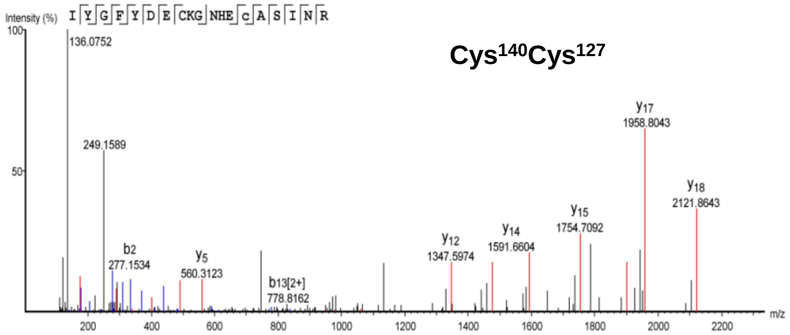

#	b	b (2+)	Seq	y	y (2+)	#
1	114.0919	57.5459	I			19
2	277.1534	139.0853	Y	2121.8643	1061.4346	18
3	334.1752	167.5883	G	1958.8043	979.9064	17
4	481.2050	241.0947	F	1901.7899	951.3989	16
5	644.3084	322.6542	Y	1754.7092	877.8610	15
6	759.3353	380.1677	D	1591.6604	796.3293	14
7	888.3779	444.6890	E	1476.6292	738.8159	13
8	991.3871	496.1935	C	1347.5974	674.2946	12
9	1119.4821	560.2410	K	1244.5800	622.7900	11
10	1176.5035	588.7659	G	1116.4851	558.7426	10
11	1290.5465	645.7733	N	1059.4636	530.2318	9
12	1427.6053	714.3027	H	945.4207	473.2103	8
13	1556.6479	778.8162	E	808.3618	404.6809	7
14	1675.6521	838.3458	C(+15.99)	679.3192	340.1631	6
15	1746.6892	873.8446	A	560.3123	280.6575	5
16	1833.7212	917.3606	S	489.2759	245.1390	4
17	1946.8053	973.9026	I	402.2433	201.6230	3
18	2060.8481	1030.9241	N	289.1609	145.0809	2
19			R	175.1183	88.0595	1

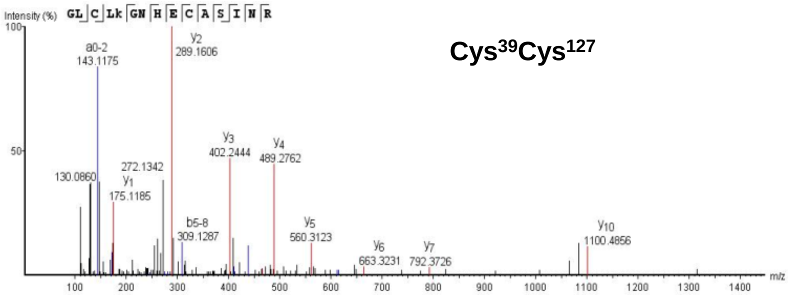

#	b	b (2+)	Seq	y	y (2+)	#
1	58.0293	29.5146	G			15
2	171.1124	86.0567	L	1573.7524	787.3762	14
3	274.0923	137.5613	C	1460.6683	730.8342	13
4	387.2066	194.1033	L	1357.6592	679.3296	12
5	531.2916	266.1458	K(+15.99)	1244.5751	622.7875	11
6	588.3130	294.6565	G	1100.4856	550.7451	10
7	702.3560	351.6780	N	1043.4686	522.2343	9
8	839.4149	420.2074	H	929.4258	465.2169	8
9	968.4575	484.7287	E	792.3726	396.6834	7
10	1071.4667	536.2333	C	663.3231	332.1621	6
11	1142.5038	571.7519	A	560.3123	280.6575	5
12	1229.5358	615.2737	S	489.2762	245.1390	4
13	1342.6199	671.8099	I	402.2444	201.6230	3
14	1456.6627	728.8314	N	289.1606	145.0809	2
15			R	175.1185	88.0595	1

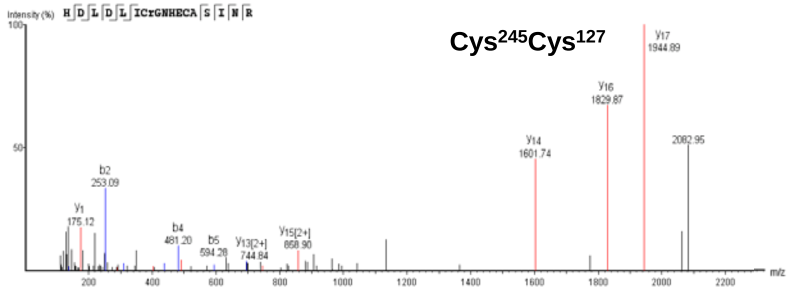

#	b	b (2+)	Seq	y	y (2+)	#
1	138.0654	69.5334	H			18
2	253.0917	127.0498	D	1944.9014	972.9507	17
3	366.1763	183.5889	L	1829.8745	915.4373	16
4	481.2001	241.1023	D	1716.7904	858.8952	15
5	594.2877	297.6443	L	1601.7634	801.3676	14
6	707.3567	354.1864	I	1488.6794	744.8397	13
7	810.3882	405.6910	C	1375.6013	688.2977	12
8	966.4831	483.7415	R	1272.5862	636.7931	11
9	1023.5045	512.2523	G	1116.4851	558.7426	10
10	1137.5475	569.2737	N	1059.4636	530.2318	9
11	1274.6064	637.8032	H	945.4207	473.2103	8
12	1403.6489	702.3245	E	808.3618	404.6809	7
13	1522.6531	761.8265	C(+15.99)	679.3192	340.1596	6
14	1593.6902	797.3451	A	560.3099	280.6575	5
15	1680.7222	840.8611	S	489.2754	245.1390	4
16	1793.8063	897.4031	I	402.2429	201.6230	3
17	1907.8492	954.4246	N	289.1606	145.0809	2
18			R	175.1183	88.0595	1

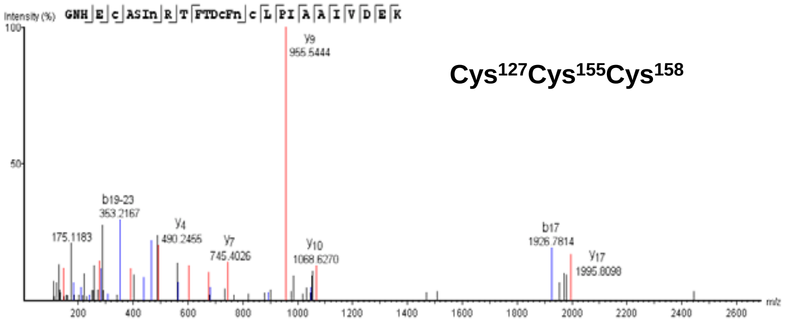

#	b	b (2+)	Seq	y	y (2+)	#
1	58.0293	29.5146	G			28
2	172.0722	86.5361	N	3088.3511	1544.6755	27
3	309.1294	155.0656	H	2974.3081	1487.6541	26
4	438.1689	219.5869	E	2837.2490	1419.1245	25
5	507.1952	254.0976	C(-33.99)	2708.2065	1354.6033	24
6	578.2323	289.6161	A	2639.1851	1320.0925	23
7	665.2643	333.1321	S	2568.1479	1284.5740	22
8	778.3484	389.6742	I	2481.1160	1241.0580	21
9	893.3432	447.1877	N(+.98)	2368.0320	1184.5160	20
10	1049.5020	525.2382	R	2253.0049	1127.0024	19
11	1150.5242	575.7621	T	2096.9038	1048.9519	18
12	1297.5925	649.2963	F	1995.8098	998.4281	17
13	1398.6401	699.8201	T	1848.7877	924.8939	16
14	1513.6671	757.3336	D	1747.7400	874.3700	15
15	1664.6611	832.8306	C(+47.98)	1632.7131	816.8566	14
16	1811.7295	906.3647	F	1481.7191	741.3596	13
17	1926.7814	963.8782	N(+.98)	1334.6508	667.8254	12
18	2077.7505	1039.3752	C(+47.98)	1219.6238	610.3119	11
19	2190.8345	1095.9172	L	1068.6270	534.8149	10
20	2287.8872	1144.4436	P	955.5444	478.2729	9
21	2400.9712	1200.9856	I	858.4930	429.7465	8
22	2472.0083	1236.5042	A	745.4026	373.2045	7
23	2543.0454	1272.0227	A	674.3690	337.6859	6
24	2656.1296	1328.5648	I	603.3309	302.1674	5
25	2755.1980	1378.0990	V	490.2455	245.6254	4
26	2870.2249	1435.6124	D	391.1812	196.0912	3
27	2999.2676	1500.1338	E	276.1541	138.5777	2
28			K	147.1118	74.0564	1

B

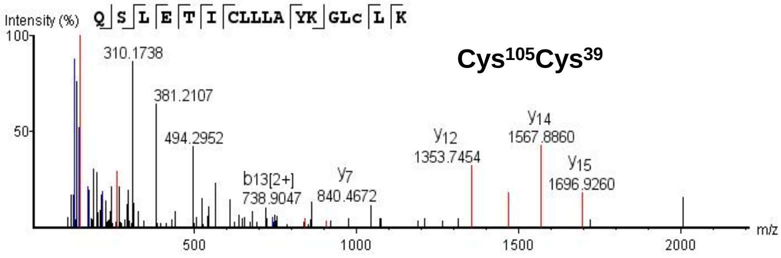

#	b	b (2+)	Seq	y	y (2+)	#
1	129.1014	65.0332	Q			18
2	216.0966	108.5492	S	1897.0537	949.0269	17
3	329.1825	165.0912	L	1810.0216	905.5018	16
4	458.2251	229.6125	E	1696.9260	848.9688	15
5	559.2728	280.1364	T	1567.8860	784.4475	14
6	672.3568	336.6784	I	1466.8484	733.9236	13
7	775.3660	388.1830	C	1353.7454	677.3816	12
8	888.4501	444.7250	L	1250.7540	625.8770	11
9	1001.5341	501.2671	L	1137.6699	569.3350	10
10	1114.6182	557.8091	L	1024.5859	512.7930	9
11	1185.6553	593.3276	A	911.5018	456.2509	8
12	1348.7186	674.8593	Y	840.4672	420.7324	7
13	1476.8136	738.9047	K	677.4014	339.2007	6
14	1533.8351	767.4175	G	540.3065	275.1532	5
15	1646.9191	823.9595	L	492.2850	246.6425	4
16	1765.9232	883.4616	C(+15.99)	379.2009	190.1005	3
17	1879.0073	940.0037	L	260.1950	130.5984	2
18			K	147.1119	74.0564	1

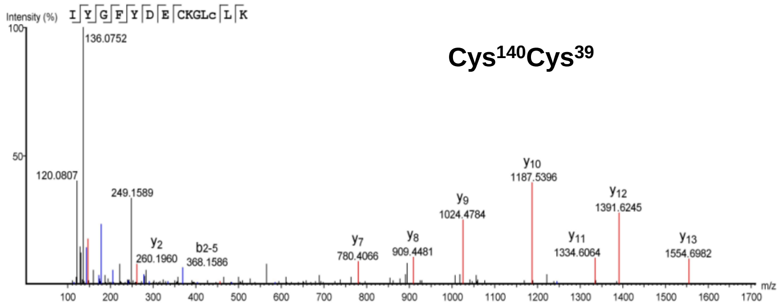

#	b	b (2+)	Seq	y	y (2+)	#
1	114.0912	57.5459	I			14
2	277.1537	139.0776	Y	1554.6982	777.8483	13
3	334.1738	167.5883	G	1391.6245	696.3167	12
4	481.2451	241.1556	F	1334.6064	667.8059	11
5	644.3084	322.6542	Y	1187.5396	594.2717	10
6	759.3353	380.1677	D	1024.4784	512.7401	9
7	888.3779	444.6890	E	909.4481	455.2284	8
8	991.3871	496.1935	C	780.4066	390.7053	7
9	1119.4821	560.2410	K	677.4014	339.2007	6
10	1176.5035	588.7518	G	549.3065	275.1532	5
11	1289.5876	645.2938	L	492.2850	246.6425	4
12	1408.5917	704.7958	C(+15.99)	379.2009	190.1005	3
13	1521.6758	761.3379	L	260.1960	130.5984	2
14			K	147.1125	74.0564	1

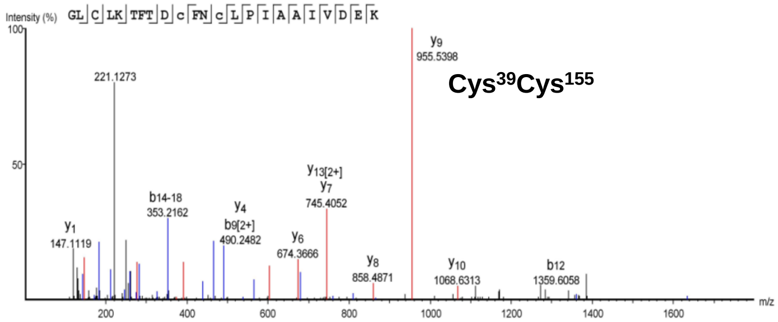

#	b	b (2+)	Seq	y	y (2+)	#
1	58.0293	29.5146	G			23
2	171.1117	86.0567	L	2530.2390	1265.6195	22
3	274.1210	137.5613	C	2417.1548	1209.0774	21
4	387.2066	194.1033	L	2314.1458	1157.5729	20
5	515.3015	258.1433	K	2201.0615	1101.0308	19
6	616.3492	308.6746	T	2072.9668	1036.9834	18
7	763.4176	382.2088	F	1971.9189	986.4595	17
8	864.4749	432.7327	T	1824.8506	912.9253	16
9	979.4922	490.2482	D	1723.8029	862.4014	15
10	1098.4963	549.7482	C(+15.99)	1608.7760	804.8880	14
11	1245.5648	623.2824	F	1489.7719	745.4052	13
12	1359.6058	680.3038	N	1342.7035	671.8517	12
13	1519.6383	760.3129	C(+57.02)	1228.6605	614.8303	11
14	1632.7354	816.8612	L	1068.6313	534.8149	10
15	1729.7751	865.3876	P	955.5398	478.2729	9
16	1842.8593	921.9296	I	858.4871	429.7465	8
17	1913.8964	957.4482	A	745.4052	373.1691	7
18	1984.9335	992.9667	A	674.3666	337.6859	6
19	2098.0176	1049.5088	I	603.3330	302.1674	5
20	2197.0859	1099.0430	V	490.2482	245.6254	4
21	2312.1128	1156.5564	D	391.1800	196.0912	3
22	2441.1555	1221.0778	E	276.1537	138.5777	2
23			K	147.1119	74.0564	1

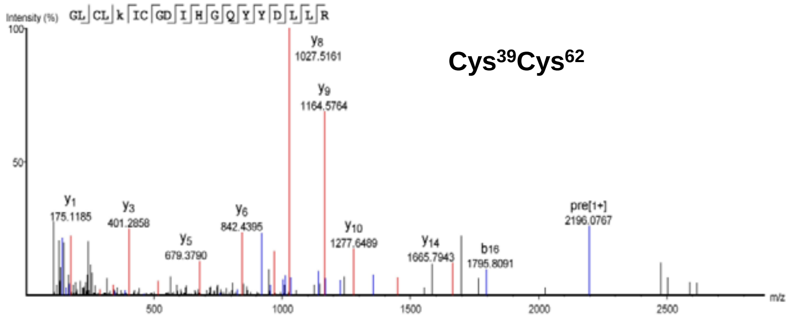

#	b	b (2+)	Seq	y	y (2+)	#
1	58.0293	29.5146	G			19
2	171.1125	86.0567	L	2139.0676	1070.0338	18
3	274.1225	137.5613	C	2025.9835	1013.4918	17
4	387.2015	194.1033	L	1922.9744	961.9872	16
5	531.2916	266.1458	K(+15.99)	1809.8903	905.4451	15
6	644.3756	322.6878	I	1665.7943	833.4026	14
7	747.3848	374.1924	C	1552.7212	776.8606	13
8	804.4062	402.7031	G	1449.7147	725.3560	12
9	919.4332	460.2167	D	1392.6906	696.8453	11
10	1032.5148	516.7586	I	1277.6489	639.3318	10
11	1169.5762	585.2881	H	1164.5764	582.7898	9
12	1226.6023	613.7988	G	1027.5161	514.2604	8
13	1354.6494	677.8281	Q	970.4938	485.7496	7
14	1517.7195	759.3667	Y	842.4395	421.7203	6
15	1680.7828	840.8914	Y	679.3790	340.1611	5
16	1795.8091	898.4049	D	516.3127	258.6570	4
17	1908.8939	954.9480	L	401.2858	201.1435	3
18	2021.9779	1011.4739	L	288.2021	144.6015	2
19			R	175.1185	88.0595	1

Figure S6. Ensemble of disulfide spectra and ion tables for Cys[39] (**B**) and Cys[127] (**A**) in GST-tagged rPP-1α. B-type ion series are indicated in blue and y-type ion series are indicated in red. The red lines indicate the hidden C-terminus of the first peptide.

A (Tyr306) His-tagged rPP-1α (-Mn^{2+} -H_2O_2)

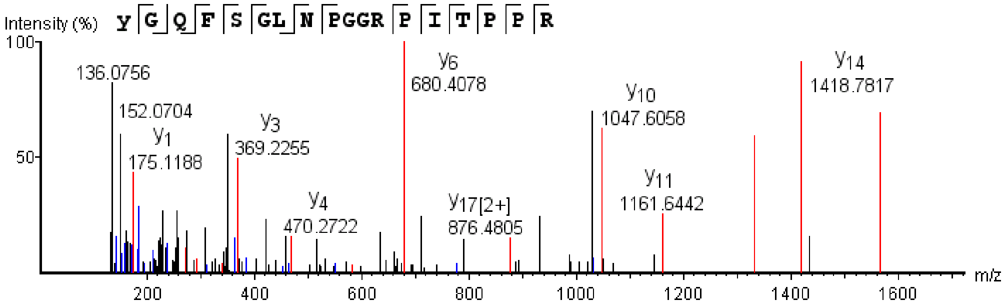

*+15.99 Da*1~15.99 Da*

#	b	b (2+)	Seq	y	y (2+)	#
1	180.0661	90.5330	Y(+15.99)			18
2	237.0860	119.0438	G	1750.9347	875.9763	17
3	365.1443	183.0730	Q	1693.9132	847.4566	16
4	512.2145	256.6073	F	1565.8563	783.4273	15
5	599.2465	300.1233	S	1418.7817	709.8931	14
6	656.2680	328.6340	G	1331.7532	666.3771	13
7	769.3521	385.1501	L	1274.7327	637.8663	12
8	883.3950	442.1975	N	1161.6442	581.3243	11
9	980.4478	490.7239	P	1047.6058	524.3029	10
10	1037.4692	519.2346	G	950.5530	475.7765	9
11	1094.4907	547.7454	G	893.5315	447.2657	8
12	1250.5918	625.7959	R	836.5101	418.7550	7
13	1347.6445	674.3223	P	680.4078	340.7061	6
14	1460.7286	730.8643	I	583.3511	292.1288	5
15	1561.7762	781.3881	T	470.2722	235.6361	4
16	1658.8290	829.9145	P	369.2255	185.1122	3
17	1755.8818	878.4409	P	272.1707	136.5858	2
18			R	175.1188	88.0595	1

B (Tyr306) His-tagged rPP-1α (-Mn^{2+} +H_2O_2)

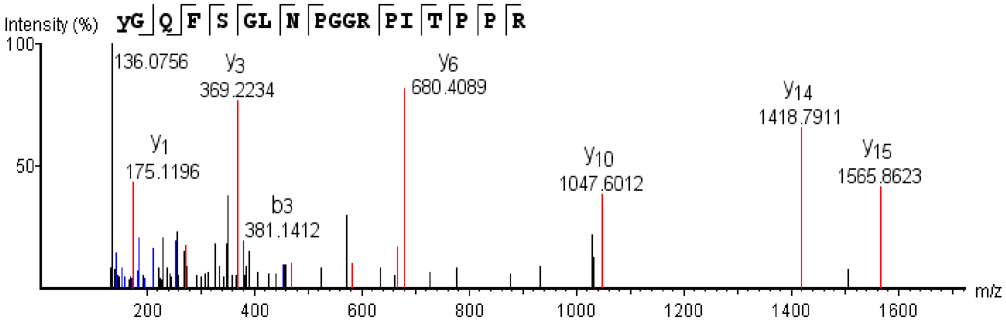

*+15.99 Da*2~31.98 Da*

#	b	b (2+)	Seq	y	y (2+)	#
1	196.0610	98.5305	Y(+31.99)			18
2	253.0815	127.0412	G	1750.9347	875.9673	17
3	381.1412	191.0705	Q	1693.9132	847.4566	16
4	528.2094	264.6047	F	1565.8623	783.4273	15
5	615.2415	308.1207	S	1418.7911	709.8931	14
6	672.2629	336.6314	G	1331.7542	666.3804	13
7	785.3469	393.1735	L	1274.7327	637.8663	12
8	899.3899	450.1949	N	1161.6487	581.3263	11
9	996.4426	498.7213	P	1047.6012	524.3029	10
10	1053.4641	527.2321	G	950.5530	475.7765	9
11	1110.4856	555.7428	G	893.5315	447.2657	8
12	1266.5867	633.7933	R	836.5101	418.7550	7
13	1363.6394	682.3197	P	680.4089	340.7045	6
14	1476.7235	738.8618	I	583.3562	292.1781	5
15	1577.7711	789.3856	T	470.2754	235.6361	4
16	1674.8240	837.9120	P	369.2234	185.1122	3
17	1771.8767	886.4384	P	272.1709	136.5858	2
18			R	175.1196	88.0595	1

C (Tyr306) GST-tagged rPP-1α (+Mn^{2+} -H_2O_2)

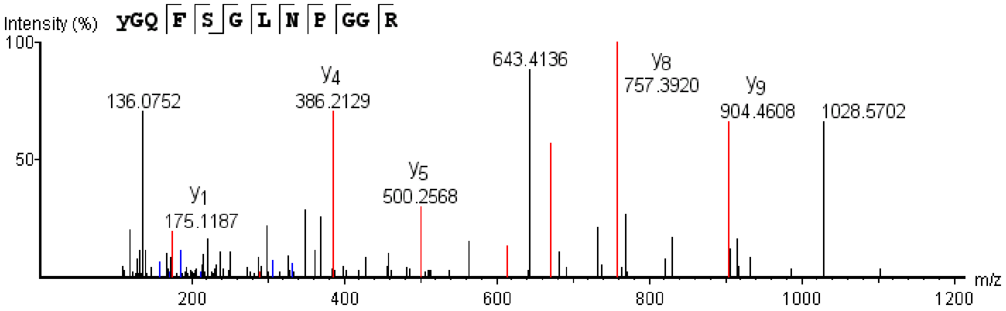

*+15.99 Da*2-2.02 Da~30.01 Da*

#	b	b (2+)	Seq	y	y (2+)	#
1	194.0817	97.5409	Y(+30.01)			12
2	251.1032	126.0516	G	1089.5436	545.2718	11
3	379.1617	190.0809	Q	1032.5221	516.7610	10
4	526.2302	263.6151	F	904.4608	452.7318	9
5	613.2621	307.1423	S	757.3920	379.1975	8
6	670.2836	335.6418	G	670.3604	335.6815	7
7	783.3677	392.1838	L	613.3397	307.1708	6
8	897.4106	449.2053	N	500.2568	250.6288	5
9	994.4634	497.7317	P	386.2129	193.6073	4
10	1051.4849	526.2424	G	289.1624	145.0809	3
11	1108.5063	554.7532	G	232.1404	116.5702	2
12			R	175.1187	88.0595	1

D (Tyr306) GST-tagged rPP-1α (+Mn^{2+} +H_2O_2)

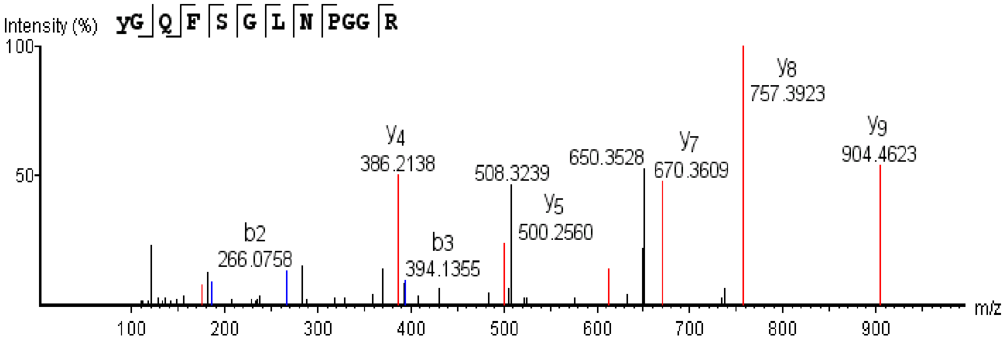

*+15.99 Da*3-3.03 Da~44.99 Da*

#	b	b (2+)	Seq	y	y (2+)	#
1	209.0562	105.0281	Y(+44.99)			12
2	266.0758	133.5388	G	1089.5436	545.2718	11
3	394.1355	197.5681	Q	1032.5221	516.7610	10
4	541.2047	271.1023	F	904.4623	452.7318	9
5	628.2367	314.6183	S	757.3923	379.1975	8
6	685.2582	343.1291	G	670.3609	335.6815	7
7	798.3422	399.6711	L	613.3399	307.1708	6
8	912.3851	456.6926	N	500.2560	250.6288	5
9	1009.4379	505.2189	P	386.2138	193.6073	4
10	1066.4594	533.7297	G	289.1619	145.0809	3
11	1123.4808	562.2404	G	232.1404	116.5702	2
12			R	175.1182	88.0595	1

Figure S7. Spectra of Tyr307 oxidations observed at the indicated conditions. (**A-B**) Spectra (left) and ion tables (right) of the normally mono- and di-oxidized Tyr307 in His-tagged rPP-1α when no Mn^{2+} was added to the buffer. (**C-D**) Spectra (left) and ion tables (right) of the unconventionally di- and tri-oxidized products of Tyr307 in GST-tagged PP-1α when Mn^{2+} is added to the buffer.

A

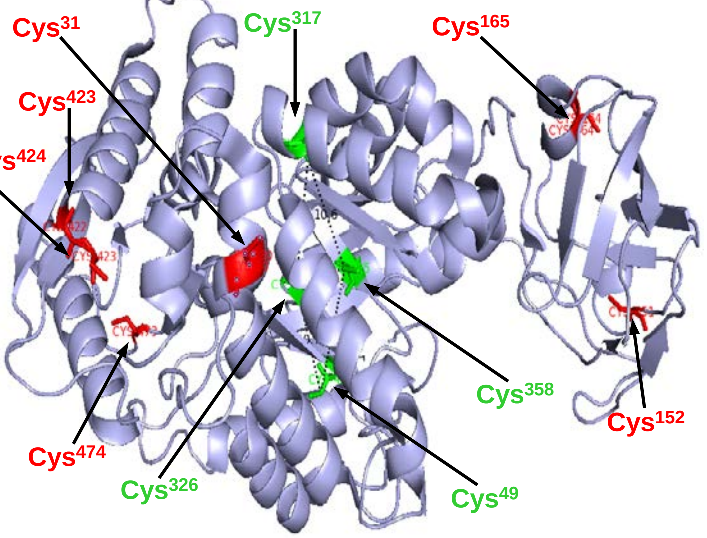

B

Cross reactivity scheme for PKM2

	Cys^{31}	Cys^{49}	Cys^{152}	Cys^{165}	Cys^{317}	Cys^{326}	Cys^{358}	Cys^{423}	Cys^{424}	Cys^{474}
Cys^{31}		58.3	57.9	56.8	30.7	26.2	31.2	28.8	22.3	47.2
Cys^{49}	58.3		38.1	42.7	20.7	10.8	10.7	32.2	28.9	26.9
Cys^{152}	57.9	38.1		15.5	38.7	35.2	40.4	65.8	62.7	60.1
Cys^{165}	56.8	42.7	15.5		35.1	35.7	41.0	64.4	62.2	61.2
Cys^{317}	30.7	20.7	38.7	35.1		10.6	10.8	29.4	28.5	31.8
Cys^{326}	26.2	10.8	35.2	35.7	10.6		7.2	31.6	27.7	34.7
Cys^{358}	31.2	10.7	40.4	41.0	10.8	7.2		25.9	24.0	24.1
Cys^{423}	28.8	32.2	65.8	64.4	29.4	31.6	25.9		7.8	28.0
Cys^{424}	22.3	28.9	62.7	62.2	28.5	27.7	24.0	7.8		24.5
Cys^{474}	47.2	26.9	60.1	61.2	31.8	34.7	24.1	28.0	24.5	

C

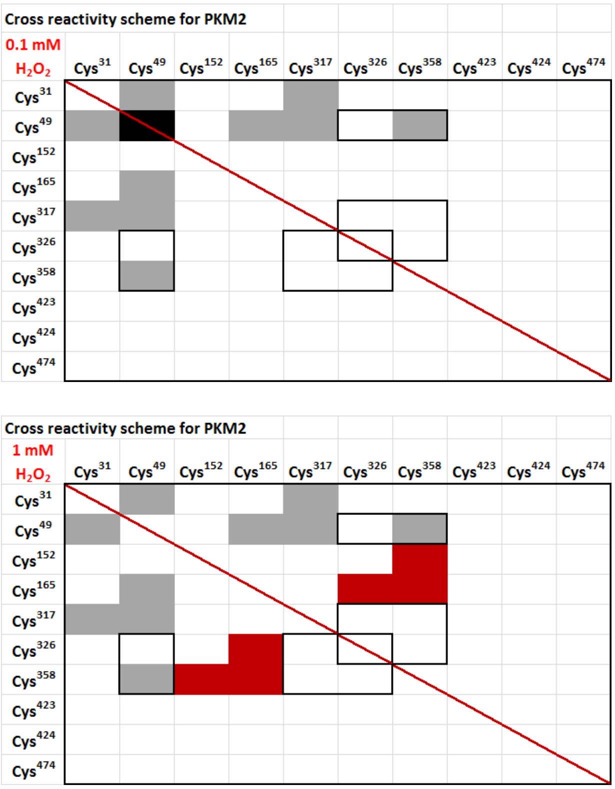

D

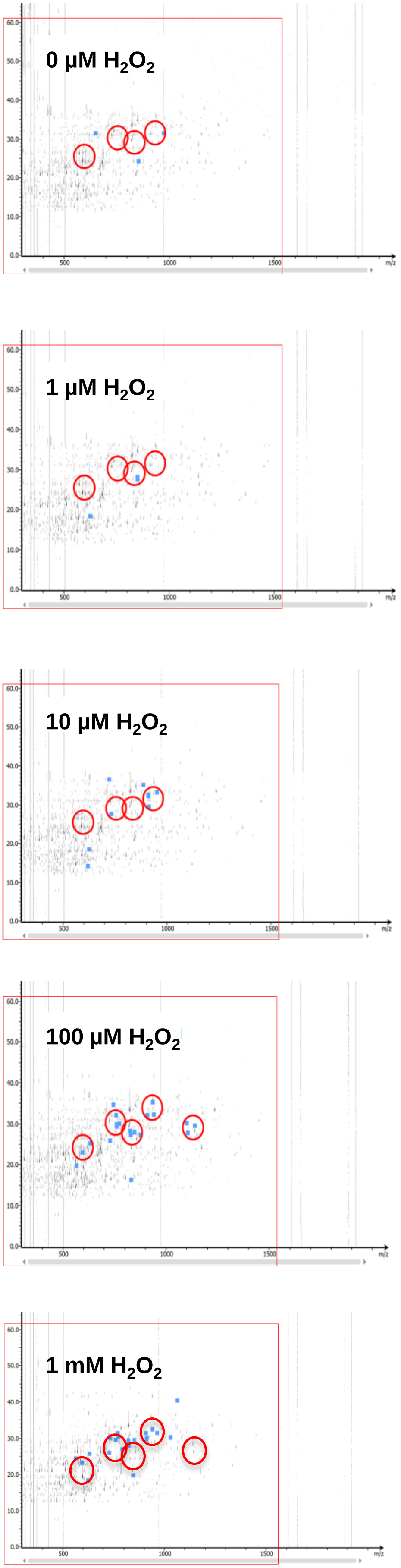

E

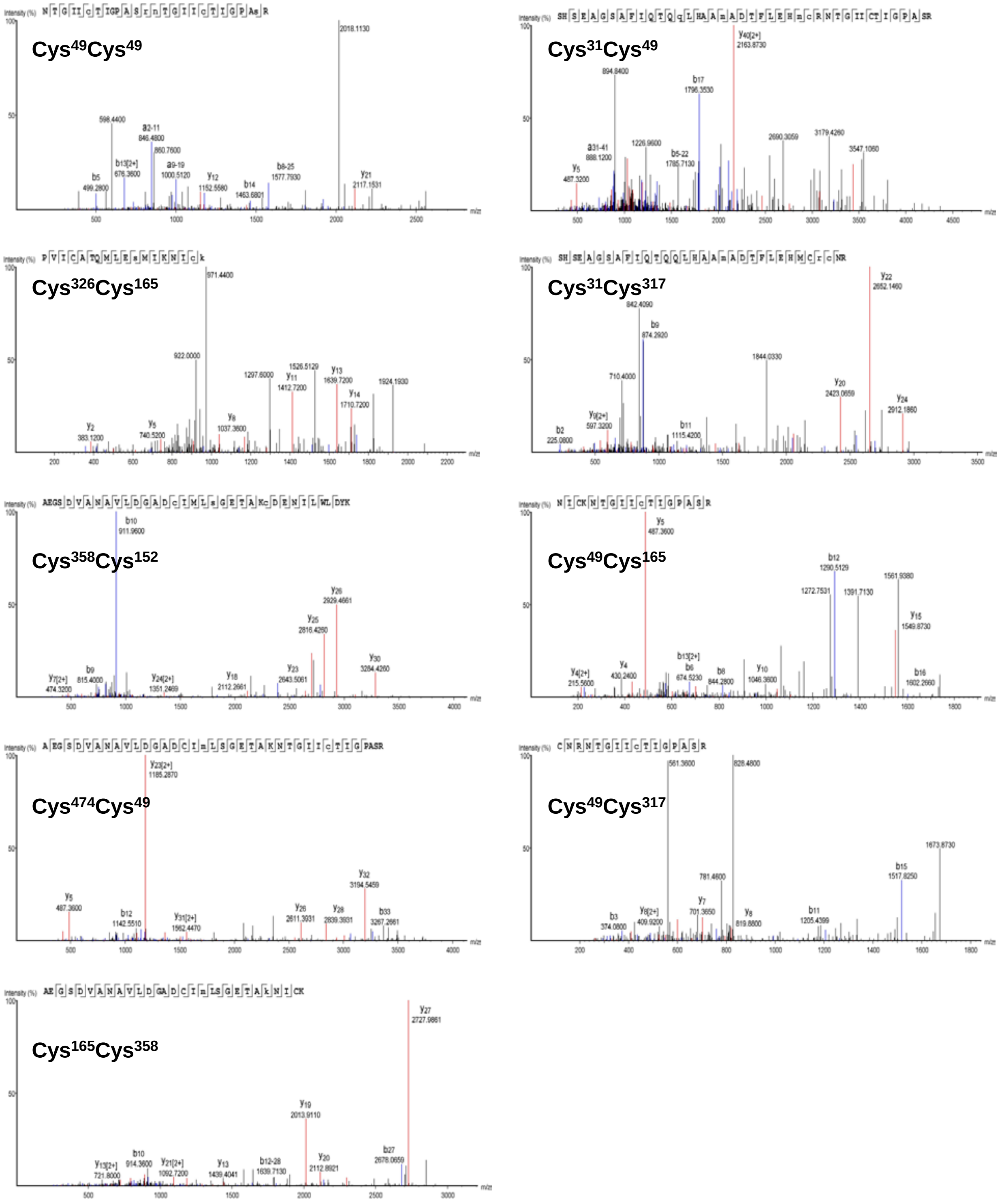

Figure S8. H_2O_2 concentration-dependent formation of transient disulfides in PKM2. (**A**) Structure plots for PKM2 was prepared using PDB-file *3srh*. Presentation of the structure is in light-blue cartoon-view, cysteine residues at the outer rim of the fold are highlighted as red sticks, cysteine residues forming the inner network are highlighted in bright green. The inner network is also zoomed-in. Dashed lines between cysteines indicate the probable network. (**B**) Cross-reactivity scheme shows the distances of all cysteines with each other. (**C**) Cross-reactivity schemes show the identified cysteine combinations at 0.1 mM and 1 mM H_2O_2. Additional disulfides appearing at higher concentration are indicated with red boxes. (**D**) Heat-maps for increasing concentrations of H_2O_2 indicate increasing numbers of predicted MS/MS-spectra with disulfides (red circles around blue dots). (**E**) Sequence views and example spectra for PKM2 generated with 1 mM H_2O_2 treatment.

Table S1. Spectral-count quantification using Peaks^{TM} of differently modified cysteines in His-tagged and GST-tagged rPP-1α. The degree of blue colour is a direct read of the percentage distribution. Green boxes indicate the identified disulfide spectra.

	GST	HIS	Mn^{2+}	H_2O_2	Cys^{39}	Cys^{62}	Cys^{105}	Cys^{127}	Cys^{140}	Cys^{155}	Cys^{158}	Cys^{171}	Cys^{172}	Cys^{202}	Cys^{245}	Cys^{273}	Cys^{291}
Propka																	
COPA																	
DiANNA																	
dbGSH																	
Oxidized PP-1 (PDB id: 4UT3)																	
Glutathionylation [%]	✓				0.00	0.00	0.00	0.00	2.63	0.00	0.00	0.00	0.00	3.95	1.32	0.00	0.00
Sulfonic acid [%]	✓				0.00	0.00	0.00	0.00	0.00	9.09	6.25	0.00	0.00	0.00	0.00	0.00	0.00
Persulfide/Dehydroalanine/Disulfide [%]	✓				100.00	0.00	0.00	100.00	33.33	27.27	18.75	30.00	36.36	0.00	25.00	25.00	0.00
Free Cysteine [%]	✓				0.00	100.00	100.00	0.00	66.67	63.64	75.00	70.00	63.64	100.00	75.00	75.00	100.00
Disulfide spectrum	✓																
Glutathionylation [%]	✓			✓	0.00	0.00	0.00	0.00	14.29	0.00	0.00	0.00	0.00	25.00	16.67	0.00	0.00
Sulfonic acid [%]	✓			✓	0.00	28.57	57.14	0.00	35.71	14.29	18.18	22.22	25.00	33.33	33.33	0.00	0.00
Persulfide/Dehydroalanine/Disulfide [%]	✓			✓	100.00	42.86	42.86	100.00	42.86	71.43	45.45	55.56	62.50	16.67	33.33	100.00	100.00
Free Cysteine [%]	✓			✓	0.00	28.57	0.00	0.00	7.14	14.29	36.36	22.22	12.50	25.00	16.67	0.00	0.00
Disulfide spectrum	✓			✓													
Glutathionylation [%]	✓		✓		0.00	0.00	0.00	0.00	0.00	0.00	0.00	0.00	0.00	0.00	0.00	0.00	0.00
Sulfonic acid [%]	✓		✓		0.00	0.00	0.00	0.00	12.50	0.00	0.00	0.00	0.00	0.00	0.00	0.00	0.00
Persulfide/Dehydroalanine/Disulfide [%]	✓		✓		100.00	0.00	0.00	100.00	37.50	50.00	25.00	20.00	12.50	0.00	0.00	0.00	0.00
Free Cysteine [%]	✓		✓		0.00	100.00	100.00	0.00	50.00	50.00	75.00	80.00	87.50	100.00	100.00	100.00	100.00
Disulfide spectrum	✓		✓														
Glutathionylation [%]	✓		✓	✓	0.00	0.00	0.00	0.00	0.00	0.00	0.00	0.00	0.00	0.00	0.00	0.00	0.00
Sulfonic acid [%]	✓		✓	✓	0.00	25.00	0.00	0.00	33.33	0.00	0.00	0.00	0.00	0.00	50.00	0.00	0.00
Persulfide/Dehydroalanine/Disulfide [%]	✓		✓	✓	0.00	0.00	0.00	0.00	0.00	100.00	50.00	66.67	66.67	0.00	0.00	0.00	0.00
Free Cysteine [%]	✓		✓	✓	0.00	75.00	0.00	0.00	66.67	0.00	50.00	33.33	33.33	0.00	50.00	0.00	0.00
Disulfide spectrum	✓		✓	✓													
Glutathionylation [%]		✓			0.00	0.00	0.00	0.00	0.00	0.00	0.00	0.00	0.00	0.00	0.00	0.00	0.00
Sulfonic acid [%]		✓			0.00	0.00	0.00	0.00	0.00	5.71	0.00	0.00	0.00	0.00	0.00	0.00	0.00
Persulfide/Dehydroalanine/Disulfide [%]		✓			0.00	0.00	0.00	0.00	0.00	45.71	48.48	42.86	42.86	0.00	0.00	0.00	0.00
Free Cysteine [%]		✓			0.00	100.00	100.00	0.00	100.00	48.57	51.52	57.14	57.14	100.00	100.00	0.00	0.00
Disulfide spectrum		✓															
Glutathionylation [%]		✓		✓	0.00	0.00	0.00	0.00	0.00	0.00	0.00	0.00	0.00	0.00	0.00	0.00	0.00
Sulfonic acid [%]		✓		✓	0.00	41.67	82.05	100.00	100.00	25.93	18.37	33.33	33.33	100.00	100.00	0.00	100.00
Persulfide/Dehydroalanine/Disulfide [%]		✓		✓	100.00	0.00	2.56	0.00	0.00	64.81	71.43	66.67	66.67	0.00	0.00	0.00	0.00
Free Cysteine [%]		✓		✓	0.00	58.33	15.38	0.00	0.00	9.26	10.20	0.00	0.00	0.00	0.00	0.00	0.00
Disulfide spectrum		✓		✓													
Glutathionylation [%]		✓	✓		0.00	0.00	0.00	0.00	0.00	0.00	0.00	0.00	0.00	0.00	0.00	0.00	0.00
Sulfonic acid [%]		✓	✓		0.00	0.00	3.33	0.00	0.00	4.44	0.00	0.00	0.00	0.00	0.00	0.00	0.00
Persulfide/Dehydroalanine/Disulfide [%]		✓	✓		0.00	0.00	0.00	0.00	0.00	53.33	55.81	35.42	35.42	0.00	0.00	0.00	0.00
Free Cysteine [%]		✓	✓		100.00	100.00	96.67	0.00	100.00	42.22	44.19	64.58	64.58	0.00	100.00	0.00	0.00
Disulfide spectrum		✓	✓														
Glutathionylation [%]		✓	✓	✓	0.00	0.00	0.00	0.00	0.00	0.00	0.00	0.00	0.00	0.00	0.00	0.00	0.00
Sulfonic acid [%]		✓	✓	✓	0.00	0.00	36.36	0.00	35.00	0.00	0.00	0.00	0.00	0.00	40.00	0.00	0.00
Persulfide/Dehydroalanine/Disulfide [%]		✓	✓	✓	0.00	0.00	0.00	0.00	0.00	70.00	70.00	62.50	62.50	0.00	0.00	0.00	0.00
Free Cysteine [%]		✓	✓	✓	100.00	100.00	63.64	0.00	75.00	30.00	30.00	37.50	37.00	0.00	60.00	0.00	0.00
Disulfide spectrum		✓	✓	✓													

Table S2. LFQ-quantification using Max Quant™ of differentially modified cysteines, to overlap the information with the spectral-count quantification from Table S1. The colour code is from green for lowest value to red for the highest value. (**A**) Quantification for GST-tagged rPP-1α. (**B**) Quantification for His-tagged rPP-1α.

A

		Free Cys			
Peptide	Cys	no Mn^{2+} no H_2O_2	no Mn^{2+} with H_2O_2	with Mn^{2+} no H_2O_2	with Mn^{2+} with H_2O_2
GLCLK	Cys^{39}	0.00E+00	0.00E+00	0.00E+00	0.00E+00
ICGDIHGQYYDLLR	Cys^{62}	1.48E+09	8.08E+07	3.45E+08	1.21E+07
QSLETICLLLAYK	Cys^{105}	0.00E+00	0.00E+00	2.40E+07	0.00E+00
GNHECASINR	Cys^{127}	0.00E+00	0.00E+00	0.00E+00	0.00E+00
IYGFYDECKR	Cys^{140}	1.95E+08	8.34E+06	0.00E+00	1.31E+06
IFCCHGGLSPDLQSMEQIR	Cys^{171}	1.62E+08	3.37E+07	3.98E+07	0.00E+00
IFCCHGGLSPDLQSMEQIR	Cys^{172}	1.62E+08	3.37E+07	3.98E+07	0.00E+00
TFTDCFNCLPIAAIVDEK	Cys^{155}	7.44E+08	2.26E+07	5.71E+06	0.00E+00
TFTDCFNCLPIAAIVDEK	Cys^{158}	7.46E+08	1.93E+07	5.71E+06	0.00E+00
PTDVPDQGLLCDLLWSDPDKDVQGWGENDR	Cys^{202}	2.34E+08	4.27E+07	2.82E+06	0.00E+00
HDLDLICR	Cys^{245}	9.62E+08	3.04E+07	4.37E+08	5.50E+06

		Sulfone Cys			
Peptide	Cys	no Mn^{2+} no H_2O_2	no Mn^{2+} with H_2O_2	with Mn^{2+} no H_2O_2	with Mn^{2+} with H_2O_2
GLCLK	Cys^{39}	0.00E+00	0.00E+00	0.00E+00	0.00E+00
ICGDIHGQYYDLLR	Cys^{62}	0.00E+00	0.00E+00	0.00E+00	0.00E+00
QSLETICLLLAYK	Cys^{105}	0.00E+00	1.08E+08	0.00E+00	0.00E+00
GNHECASINR	Cys^{127}	0.00E+00	0.00E+00	0.00E+00	0.00E+00
IYGFYDECKR	Cys^{140}	0.00E+00	1.01E+08	1.42E+06	6.28E+06
IFCCHGGLSPDLQSMEQIR	Cys^{171}	0.00E+00	9.49E+06	0.00E+00	0.00E+00
IFCCHGGLSPDLQSMEQIR	Cys^{172}	0.00E+00	9.49E+06	0.00E+00	0.00E+00
TFTDCFNCLPIAAIVDEK	Cys^{155}	0.00E+00	3.31E+06	0.00E+00	0.00E+00
TFTDCFNCLPIAAIVDEK	Cys^{158}	0.00E+00	3.31E+06	0.00E+00	0.00E+00
PTDVPDQGLLCDLLWSDPDKDVQGWGENDR	Cys^{202}	0.00E+00	1.07E+07	0.00E+00	0.00E+00
HDLDLICR	Cys^{245}	0.00E+00	6.07E+07	0.00E+00	5.17E+06

		GSH Cys			
Peptide	Cys	no Mn^{2+} no H_2O_2	no Mn^{2+} with H_2O_2	with Mn^{2+} no H_2O_2	with Mn^{2+} with H_2O_2
GLCLK	Cys^{39}	0.00E+00	0.00E+00	0.00E+00	0.00E+00
ICGDIHGQYYDLLR	Cys^{62}	0.00E+00	0.00E+00	0.00E+00	0.00E+00
QSLETICLLLAYK	Cys^{105}	0.00E+00	0.00E+00	0.00E+00	0.00E+00
GNHECASINR	Cys^{127}	0.00E+00	0.00E+00	0.00E+00	0.00E+00
IYGFYDECKR	Cys^{140}	0.00E+00	2.60E+07	0.00E+00	0.00E+00
IFCCHGGLSPDLQSMEQIR	Cys^{171}	0.00E+00	0.00E+00	0.00E+00	0.00E+00
IFCCHGGLSPDLQSMEQIR	Cys^{172}	0.00E+00	0.00E+00	0.00E+00	0.00E+00
TFTDCFNCLPIAAIVDEK	Cys^{155}	0.00E+00	0.00E+00	0.00E+00	0.00E+00
TFTDCFNCLPIAAIVDEK	Cys^{158}	0.00E+00	0.00E+00	0.00E+00	0.00E+00
PTDVPDQGLLCDLLWSDPDKDVQGWGENDR	Cys^{202}	0.00E+00	9.32E+06	0.00E+00	0.00E+00
HDLDLICR	Cys^{245}	0.00E+00	4.25E+06	0.00E+00	0.00E+00

		Dehydroalanine Cys			
Peptide	Cys	no Mn^{2+} no H_2O_2	no Mn^{2+} with H_2O_2	with Mn^{2+} no H_2O_2	with Mn^{2+} with H_2O_2
GLCLK	Cys^{39}	0.00E+00	0.00E+00	0.00E+00	0.00E+00
ICGDIHGQYYDLLR	Cys^{62}	0.00E+00	0.00E+00	0.00E+00	0.00E+00
QSLETICLLLAYK	Cys^{105}	0.00E+00	3.56E+06	0.00E+00	0.00E+00
GNHECASINR	Cys^{127}	0.00E+00	0.00E+00	0.00E+00	0.00E+00
IYGFYDECKR	Cys^{140}	0.00E+00	0.00E+00	0.00E+00	0.00E+00
IFCCHGGLSPDLQSMEQIR	Cys^{171}	1.46E+07	0.00E+00	0.00E+00	0.00E+00
IFCCHGGLSPDLQSMEQIR	Cys^{172}	1.46E+07	0.00E+00	0.00E+00	0.00E+00
TFTDCFNCLPIAAIVDEK	Cys^{155}	1.97E+06	0.00E+00	0.00E+00	0.00E+00
TFTDCFNCLPIAAIVDEK	Cys^{158}	1.97E+06	0.00E+00	0.00E+00	0.00E+00
PTDVPDQGLLCDLLWSDPDKDVQGWGENDR	Cys^{202}	0.00E+00	0.00E+00	0.00E+00	0.00E+00
HDLDLICR	Cys^{245}	0.00E+00	0.00E+00	0.00E+00	0.00E+00

Color code
1.48E+09
8.00E+08
5.00E+08
1.00E+08
5.00E+07
1.00E+07
5.00E+06
1.00E+06
1.00E+05
1.00E+04
1.00E+03

B

Peptide	Cys	Free Cys: no Mn^{2+} no H_2O_2	Free Cys: no Mn^{2+} with H_2O_2	Free Cys: with Mn^{2+} no H_2O_2	Free Cys: with Mn^{2+} with H_2O_2	Color code
ICGDIHGQYYDLLR	Cys^{62}	1.36E+06	2.73E+05	2.89E+06	8.04E+05	1.60E+07
QSLETICLLLAYK	Cys^{105}	6.01E+06	4.85E+05	1.60E+07	2.09E+06	8.50E+06
GKQSLETICLLLAYK	Cys^{105}	3.13E+06	1.00E+03	2.07E+06	1.00E+03	5.00E+06
GNHECASINR	Cys^{127}	6.10E+04	2.68E+05	1.00E+03	1.00E+03	1.00E+06
IYGFYDECKRR	Cys^{140}					5.00E+05
TFTDCFNCLPIAAIVDEK.I	$Cys^{155}Cys^{158}$	3.02E+06	1.00E+03	3.61E+06	2.64E+05	1.00E+05
IFCCHGGLSPDLQSM(+15.99)EQIR	$Cys^{171}Cys^{172}$	1.32E+06	1.00E+03	4.60E+06	9.20E+05	5.00E+04
IFCCHGGLSPDLQSMEQIRR	$Cys^{171}Cys^{172}$	9.19E+05	1.00E+03	5.96E+06	2.27E+05	1.00E+04
IM(+15.99)RPTDVPDQGLLCDLLWSDPDK	Cys^{202}					5.00E+03
FLHKHDLDLICR	Cys^{245}	3.98E+06	1.44E+05	3.49E+06	1.00E+03	2.00E+03
HDLDLICR	Cys^{245}	1.93E+06	1.00E+03	1.16E+07	8.37E+05	1.00E+03

Peptide	Cys	Sulfone Cys: no Mn^{2+} no H_2O_2	Sulfone Cys: no Mn^{2+} with H_2O_2	Sulfone Cys: with Mn^{2+} no H_2O_2	Sulfone Cys: with Mn^{2+} with H_2O_2
ICGDIHGQYYDLLR	Cys^{62}	1.00E+03	1.09E+06	1.00E+03	1.00E+03
QSLETICLLLAYK	Cys^{105}	7.10E+04	8.40E+06	1.00E+03	1.46E+05
GKQSLETICLLLAYK	Cys^{105}	1.00E+03	3.73E+06	1.00E+03	3.67E+04
GNHECASINR	Cys^{127}				
IYGFYDECKRR	Cys^{140}	1.00E+03	7.30E+06	1.82E+05	2.13E+05
TFTDCFNCLPIAAIVDEK.I	$Cys^{155}Cys^{158}$	1.00E+03	2.41E+05	6.40E+04	1.00E+03
IFCCHGGLSPDLQSM(+15.99)EQIR	$Cys^{171}Cys^{172}$	1.00E+03	8.27E+05	1.00E+03	1.00E+03
IFCCHGGLSPDLQSMEQIRR	$Cys^{171}Cys^{172}$				
IM(+15.99)RPTDVPDQGLLCDLLWSDPDK	Cys^{202}	1.00E+03	2.96E+05	1.00E+03	1.00E+03
FLHKHDLDLICR	Cys^{245}	1.00E+03	1.01E+06	1.00E+03	1.00E+03
HDLDLICR	Cys^{245}	1.00E+03	1.98E+06	4.17E+05	5.51E+05
